# ImPROving TB outcomes by modifying LIFE-style behaviours through a brief motivational intervention followed by short text messages (ProLife): study protocol for a randomised controlled trial

**DOI:** 10.1186/s13063-019-3551-9

**Published:** 2019-07-26

**Authors:** Andrew Stephen Moriarty, Goedele Maria Louwagie, Noreen Dadirai Mdege, Neo Morojele, John Tumbo, Olufemi Babatunde Omole, Max Oscar Bachmann, Mona Kanaan, Astrid Turner, Steve Parrott, Kamran Siddiqi, Olalekan Abdulwahab Ayo-Yusuf

**Affiliations:** 1 Department of Health Sciences and the Hull York Medical School, University of York, York, UK; 2 School of Health Systems and Public Health, University of Pretoria and Sefako Makgatho Health Sciences University, Ga-Rankuwa, South Africa; 3 Department of Health Sciences, University of York, York, UK; 4 Alcohol, Tobacco and Other Drug Research Unit, South African Medical Research Council, Cape Town, South Africa; 5 Department of Family Medicine, Sefako Makgatho Health Sciences University, Ga-Rankuwa, South Africa; 6 Department of Family Medicine, University of Witwatersrand, Johannesburg, South Africa; 7 Norwich Medical School, University of East Anglia, Norwich, UK; 8 School of Health Systems and Public Health, University of Pretoria, Pretoria, South Africa; 9 Africa Centre for Tobacco Industry Monitoring and Policy Research, Sefako Makgatho Health Sciences University, Ga-Rankuwa, South Africa

**Keywords:** Tuberculosis, Smoking, Alcohol, Motivational interviewing, Anti-retroviral therapy, Adherence

## Abstract

**Background:**

South Africa is among the seven highest tuberculosis (TB) burden countries. Harmful lifestyle behaviours, such as smoking and alcohol, and poor adherence to medication can affect clinical outcomes. Modification of these behaviours is likely to improve TB treatment outcomes and has proven possible using motivational interviewing (MI) techniques or use of short message service (SMS) text messaging. There have been no studies assessing the effect of combined MI and SMS interventions on multiple lifestyle factors and TB treatment outcomes.

**Methods:**

This is a prospective, multicentre, two-arm individual randomised controlled trial looking at the effectiveness and cost-effectiveness of a complex behavioural intervention (the ProLife programme) on improving TB and lifestyle-related outcomes in three provinces of South Africa. The ProLife programme consists of an MI counselling strategy, delivered by lay health workers, augmented with subsequent SMS. We aim to recruit 696 adult participants (aged 18 years and over) with drug-sensitive pulmonary TB who are current smokers and/or report harmful or hazardous alcohol use. Patients will be consecutively enrolled at 27 clinics in three different health districts in South Africa. Participants randomised individually to the intervention arm will receive three MI counselling sessions one month apart. Each MI session will be followed by twice-weekly SMS messages targeting treatment adherence, alcohol use and tobacco smoking, as appropriate. We will assess the effect on TB treatment success, using standard World Health Organization (WHO) treatment outcome definitions (primary outcome), as well as on a range of secondary outcomes including smoking cessation, reduction in alcohol use, and TB medication and anti-retroviral therapy adherence. Secondary outcomes will be measured at the three-month and six-month follow-ups.

**Discussion:**

This trial aligns with the WHO agenda of integrating TB care with the care for chronic diseases of lifestyle, such as provision of smoking cessation treatments, and with the use of digital technologies. If the ProLife programme is found to be effective and cost-effective, the programme could have significant implications for TB treatment globally and could be successfully implemented in a wide range of TB treatment settings.

**Trial registration:**

ISRCTN Registry, ISRCTN62728852. Registered on 13 April 2018.

**Electronic supplementary material:**

The online version of this article (10.1186/s13063-019-3551-9) contains supplementary material, which is available to authorized users.

## Background

South Africa has the second highest incidence of tuberculosis (TB) and the sixth highest TB burden of the 30 high TB burden countries. It also has a high prevalence of human immunodeficiency virus (HIV) co-infection in patients with TB and a high mortality in these co-infected patients relative to most other high-burden countries [1]. In addition to HIV co-infection, a range of social, psychological and economic factors influence treatment success in TB. Of these, tobacco smoking and hazardous or harmful alcohol use are specifically mentioned in the South Africa National TB Management Guidelines [2]. Tobacco use and problem drinking are prevalent in TB patients, often co-occur [3–5] and are known to increase the risk of death and poor treatment adherence and outcomes [6–9].

As well as increasing the risk of TB infection and progression of disease [10], smoking is also known to increase the risk of treatment failure [11], relapse [12, 13] and death [14], and is associated with resistance to isoniazid, one of the main antimicrobials used to treat TB [15]. There is evidence that patients who stop smoking have better TB treatment outcomes and, if co-infected, better HIV treatment outcomes than current smokers [16–18]. In addition, alcohol misuse is associated with poorer TB outcomes through a range of mechanisms including decreased effectiveness of medications used to treat TB (including drug resistance), increased recurrence and treatment default rates and social marginalisation [3]. It has been estimated that 10% of the global burden of TB is attributable to alcohol use [19, 20].

Poor adherence to TB medication and anti-retroviral therapy (ART) significantly increases the risk of adverse effects and death in TB patients. Strategies are needed to improve medication adherence in TB-HIV co-infected patients, in whom integration of TB and HIV care has been shown to decrease mortality [21].

A number of studies have attempted to evaluate the effectiveness of tobacco cessation, alcohol reduction or TB treatment adherence interventions in TB patients [21, 22], but few have assessed the effect on TB treatment outcomes [23, 24]. We know that brief smoking cessation interventions, ranging from opportunistic advice and proactive telephone support through to in-person behavioural support over multiple sessions, are effective and affordable in low-income countries, in both TB patients and smokers in the general population [25, 26]. We also know that disease diagnosis could constitute a ‘teachable moment’, when people are more amenable to advice and motivated to modify harmful lifestyle behaviours [27]. For this reason, it is likely that patients receiving a TB diagnosis may be more successful in quitting smoking and moderating alcohol use if offered behavioural support, compared with smokers and problem drinkers in the general population.

Motivational interviewing (MI) is a counselling technique known to facilitate behaviour change [28], which has demonstrated effectiveness for the reduction of hazardous drinking, tobacco cessation, and TB treatment and/or ART adherence [19, 29, 30]. Our group has had previous success in achieving sustained smoking abstinence in TB patients through a brief MI intervention delivered by lay health workers (LHWs) [31]. There is also evidence to suggest that adherence to ART [32] and possibly TB medication [33], as well as tobacco cessation, can be enhanced with the use of short message service (SMS) text messages on mobile phone technology [34]. To our knowledge, no study has used MI in combination with SMS text messages to address multiple harmful behaviours that adversely impact TB outcomes.

The ProLife programme is a novel complex behavioural intervention targeting tobacco smoking, problem drinking, and TB and HIV medication adherence in patients with TB. This is the study protocol for the randomised controlled trial (RCT) aiming to assess the effectiveness and cost-effectiveness of the ProLife programme in improving TB treatment outcomes in primary healthcare clinics located in high TB-burden communities in three provinces in South Africa (Gauteng, Free State and North West). The protocol has been written in accordance with the Standard Protocol Items: Recommendations for Intervention Trials (SPIRIT) guideline.

## Methods/Design

### Objectives

The primary objective is to assess the effectiveness and cost-effectiveness of the ProLife programme compared to usual care in improving pulmonary TB (PTB) treatment outcomes. We also aim to assess the effectiveness and cost-effectiveness of the programme in achieving abstinence from smoking, reducing harmful and hazardous drinking, and improving TB and ART medication adherence.

### Trial design and setting

This is a pragmatic, prospective, multicentre, two-arm, parallel, individual RCT taking place in 27 purposively selected primary care clinics with the highest TB case-load in three districts in South Africa: Welkom in the Free State; Bojanala in the North West province; and Sedibeng in Gauteng province. To be eligible for inclusion in the trial, TB clinics had to be under the control of the provincial or local government (i.e. not a mining TB clinic). The intervention will be delivered by LHWs and three district coordinators who will each cover 1–2 clinics.

Participants will be randomly allocated to one of two arms:

- Arm 1: Intervention arm – participants in the interventions arm will receive the ProLife programme;
- Arm 2: Control arm – participants will receive usual treatment and support provided to TB patients in TB treatment clinics in South Africa (‘usual care’).

### Intervention

The ProLife programme is a complex behavioural intervention aimed at improving treatment outcomes in TB patients who smoke tobacco, drink alcohol at harmful or hazardous levels, or do both. The ProLife programme has been developed in line with the Medical Research Council (MRC) guidance on developing and evaluating complex interventions [35] and has undergone a period of development and feasibility testing in South Africa. The paper reporting the results of this evaluation has not yet been published. The conceptual framework used to develop the ProLife programme assumes that smoking cessation, reducing harmful alcohol use and improved adherence to TB and HIV treatment will result in improved TB treatment outcomes.

Participants randomised to the intervention arm will receive three brief MI counselling sessions, lasting 15–20 min, each one month apart, from a trained LHW at their TB clinic. In the initial MI session, occurring at the start of TB treatment, the counsellor will establish the participant’s tobacco smoking habits and problem drinking; potential obstacles and facilitators for medication adherence or treatment initiation (both TB treatment and ART) will be determined. The participant will determine which factor should be prioritised (i.e. agenda setting), which could be a plan either to quit tobacco smoking, reduce or quit drinking or deal with barriers relating to ART or TB medication adherence. For participants who are HIV-infected and not yet on ART, beliefs and attitudes regarding HIV-testing or ART will be explored to facilitate ART initiation and adherence. The second session will build on the previous one and will focus on the previous agenda before moving on to the next behavioural problem (tobacco, alcohol or medication adherence) where applicable. The third session will deal with the last identified problem.

The individual counselling sessions will be re-enforced with SMS text messages regarding information, motivation and behaviours (IMB) supporting tobacco cessation, alcohol use and medication adherence. Text messages will be delivered twice a week over 12 weeks. All participants will first receive 10 TB-related messages. These messages will be followed by seven alcohol or smoking-related messages depending on whether the participant smokes or drinks. Co-joint users will receive all sets of messages (i.e. 24 in total).

### Comparator

Participants in the second group are the controls and will receive usual TB treatment and support offered to TB patients. Control participants are seen by a TB nurse and receive the same biochemical investigations and medical treatments as the intervention arm. However, they will not receive the MI and SMS package of care as described above. Usual care also includes HIV testing with pre- and post-test HIV test counselling by a lay counsellor or a nurse (this varies by district).

In addition, health education is given on:

- nature of TB, treatment adherence, treatment side-effects/complications, drug interactions, tobacco use, alcohol use and other substance abuse. This is mostly done by the TB nurses, is not intensive and is educational in nature rather than a form of counselling;
- healthy diet by a dietician where possible;
- social problems and family support for treatment by a social worker as needed and depending on the availability of social workers; and
- point of care blood glucose, haemoglobin and pregnancy tests are performed. If co-infected with HIV, a full blood count, liver function test and creatinine are also carried out.

### Participants

The inclusion criteria for participants are:

- adult patients (aged ≥ 18 years) with drug-sensitive (bacteriologically or clinically confirmed) PTB;
- initiating TB treatment or on TB treatment for < 1 month (these include both ‘new’ and ‘retreatment’ patients);
- current smokers and/or hazardous/harmful drinkers who are not alcohol dependent (Alcohol Use Disorders Identification Test [AUDIT] score ≥ 8 for men or ≥ 7 for women but < 20);
- access to a functional mobile phone; and
- understands one of the four languages used for the trial (Sesotho, Setswana, Isizulu or English).

Exclusion criteria:

- alcohol-dependent participants (AUDIT score ≥ 20);
- Extrapulmonary TB without PTB; or
- Resistance to one or more TB drugs at baseline (because drug-resistant TB needs further investigations which take several months and is treated through a long, specialised treatment programme if present).

### Recruitment process

Participants will be recruited consecutively from the starting date of the trial until the required sample size for each clinic has been achieved. The sample size per clinic will vary depending on the workload for each clinic. TB patients who initiate treatment (or who have been on treatment for < 1 month) will be asked if they would like to participate. If they agree, they will then be screened for eligibility by trained fieldworkers immediately after the TB nurse at the clinic has initiated TB treatment and started the routinely used TB treatment record. Eligibility screening will involve being assessed in line with the inclusion criteria listed above and being asked about their:

- smoking status: using the Global Adult Tobacco Survey [36] questionnaire, patients will be asked whether they currently smoke daily, less than daily or not at all, and in the past daily, less than daily or not at all. As TB patients in South Africa often smoke little or may not have smoked for a few days because of ill-health and the word ‘current’ is open to interpretation (particularly when translated); ‘current’ has been defined as smoking daily and non-daily in the last four weeks for the purpose of this study. Smoking habits will be further quantified at baseline interview;
- current alcohol usage: AUDIT, a validated tool for identifying problem alcohol behaviours [37], will be used to quantify alcohol intake. Those with an AUDIT score ≥ 8 for men or ≥ 7 for women but < 20 will be eligible for the trial.

If eligible, patients will be invited to join the trial. For those who wish to be enrolled into the trial, written informed consent will be obtained (Additional file 1).

### Randomisation and allocation

Patients will be randomised using a randomised sequence generator performed by the trial statistician (MK) who will remain blind to the arm allocation. We will use block randomisation with varying block sizes stratified by the clinic to achieve equal numbers in intervention and control groups within each clinic (see the ‘Sample size’ section). Allocation concealment will be done with consecutively numbered, sealed, opaque envelopes.

### Ethics and consent

Potential participants will be approached and given an introduction to the study and basic eligibility criteria required for participation. Participants who meet the eligibility criteria for language, age, PTB, treatment duration and mobile phone access will be given a consent form (Additional file 1) to sign before screening for alcohol and tobacco use eligibility as the alcohol-related questions are sensitive and the fieldworkers must access the patient treatment record. If recruited to the trial, participants will be ask to consent to enter the trial, including consent for audio recording of the MI counselling sessions (Additional file 1). A witness signature will be required where the participant is unable to read or write.

The identity of participants will be protected by allocating each participant a unique trial number, which will be used on all research documents and will ensure anonymity for the data analysis. Participation will be voluntary; participants will be informed of their right to withdraw from the study at any time without giving a reason for their withdrawal. The project is unlikely to be directly harmful to TB patients with the exception of inconvenience in terms of time spent on the counselling sessions. Participants will receive ZAR 60 (US$4.19) travel and other expenses reimbursement for each MI counselling session and follow-up visit related to the study.

### Timeline and procedures

**Fig. 1:**
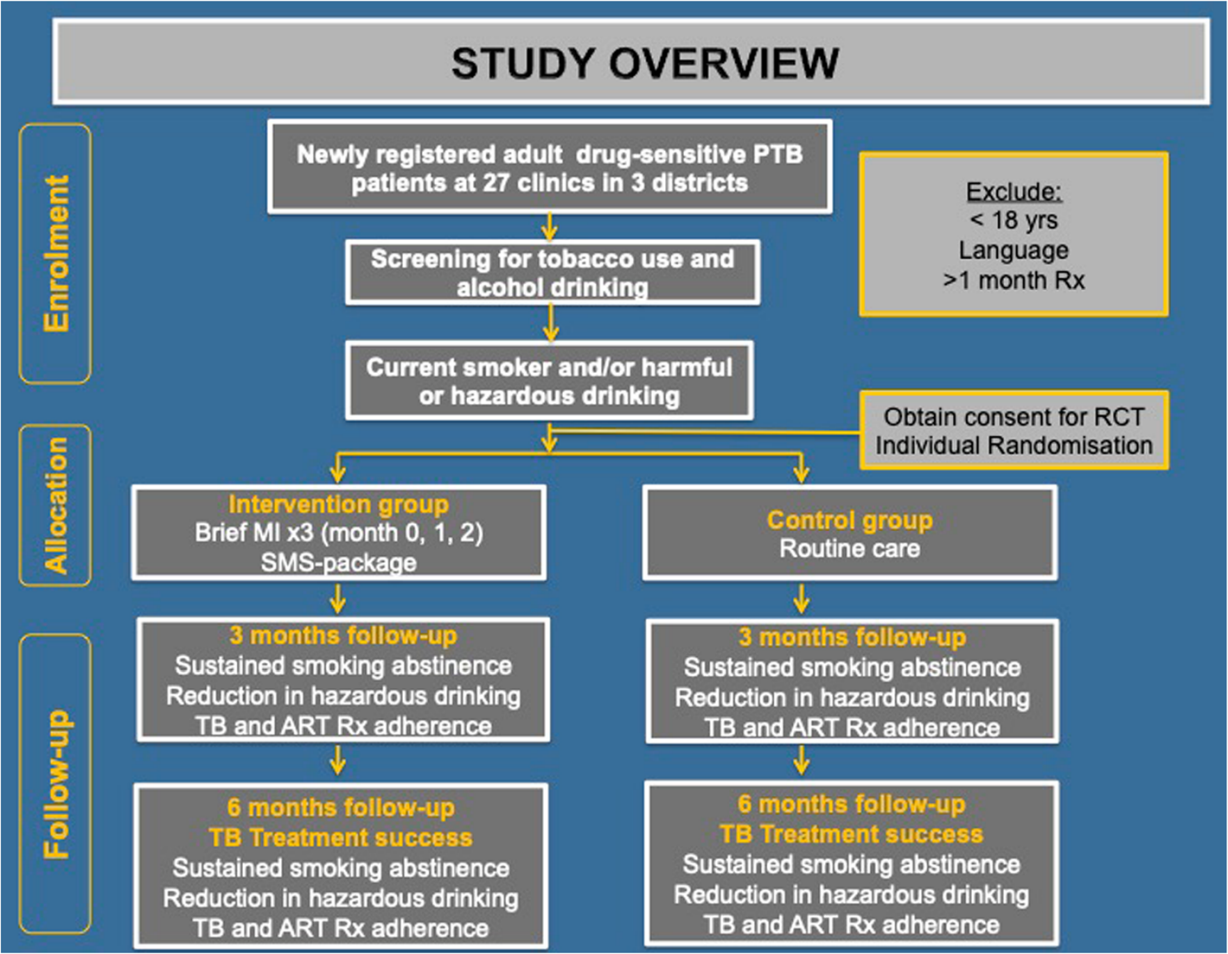
CONSORT Flow diagram outlining randomisation and participant recruitment

Figure 1 Additional file 4 outlines the overall participant flow through the trial. Eligible and consenting patients will be enrolled in the trial and the baseline interviewer-administered questionnaire and record review completed. Participants will receive a baseline interview on the same day as the recruitment or on the nearest available date suitable to both the participant and the LHW. The initial part of the questionnaire covers socioeconomic and demographic factors. We will also assess for depression using the CES-D10 [38], a validated tool, as depression may be a potentially moderating or mediating factor affecting treatment outcome. See Additional file 2 for the full case report form (CRF).

The next part will cover medical history, particularly TB and HIV history and current medications. Participants identified as current smokers at eligibility screening will be asked more in-depth about their smoking habits and quit history. All participants will be asked about their use of smokeless tobacco and exposure to second-hand smoke. Participants will also be asked about time and money spent on TB-related healthcare visits in the past three months and health-related quality of life (see the ‘Economic evaluation’ section). After administering the baseline interviews, fieldworkers will draw the allocation envelopes and organise the follow-up sessions for the participants. Each of the interviews on health status, tobacco and alcohol use at baseline, three months and six months is not expected to last > 25 min.

### Primary outcome

The primary outcome is TB treatment success at 6–9 months of follow-up, measured using the routinely collected programmatic TB treatment outcomes as defined by the World Health Organization (WHO) and adopted in South Africa. This is a binary variable defined as either successful treatment (cured or treatment completed) or failed treatment, death, acquired drug resistance, loss to follow-up or ‘default’, or not outcome evaluated. Table 1 defines the different treatment outcomes according to the South African Department of Health National Tuberculosis Management Guidelines [2].

**Table 1:** Definitions for TB treatment outcomes according to the South African Department of Health National Tuberculosis Management Guidelines

Treatment outcome	Definition
Cure	Patient in whom baseline smear or culture was positive at beginning of treatment AND is smear/culture negative in the last month of treatment and on at least one previous occasion at least 30 days prior According to local protocol, a patient who is diagnosed using Gene Xpert and is sputum negative for TB at 11 and 23 weeks is considered ‘Cured’.
Treatment completed	Patient whose baseline smear or culture was positive at the beginning and has completed treatment but does not have a negative smear/culture in the last month of treatment and on at least one previous occasion > 30 days prior. Patients diagnosed with PTB whose baseline smear (or culture) result was negative and who started treatment based on clinical and radiological findings who have shown clinical improvement and completed the prescribed course of treatment. *N.B. The smear examination may not have been done or the results may not be available at the end of treatment.*
Treatment failure	Patient whose baseline smear or culture was positive and remains or becomes positive again at 5 months or later during treatment. Patients who were negative at baseline but were later found to be positive. *N.B. This definition excludes those patients who are diagnosed with RR-TB or MDR-TB during treatment.*
Died	Patient who dies for any reason during the course of TB treatment.
Treatment default	Patient whose treatment was interrupted for two consecutive months or more during the treatment period.
Transfer out	Patient who was referred to a facility in another district to continue treatment and for whom the treatment outcome is not known.
Acquired resistance	Participants who are subsequently referred for MDR treatment.

Individual TB treatment records will be used as the primary source of information. The TB treatment record is the routinely used clinical document initiated by the TB nurse when the patient first presents at the TB clinic and includes demographic information as well as a comprehensive overview of the patient’s treatment over time. The TB treatment outcomes will be obtained 6–9 months after the TB treatment start dates. This is to allow for short periods of treatment interruption and time needed to confirm the TB treatment outcomes status. For example, the TB nurse may have to wait for end of treatment TB sputum or culture results or time may be required to determine whether a participant died. In some cases, TB patients may also undergo a longer TB treatment regimen.

Routinely reported outcomes will be verified for correctness by checking the actual individual diagnosis and treatment records. For example: if the outcome is classified as cured, then the criteria for diagnosis and outcome definitions should have been adhered to. Attempts will be made to verify the information of patients classified as ‘defaulted’ or who are lost to follow-up by contacting them telephonically or sending them a short text message. Sputum cultures and smears will be performed at baseline, two months, three months and six months per routine care.

### Secondary outcomes

The following outcome measures will also be recorded at the six-month follow-up:

- sputum conversion at the end of treatment: this will be measured by negative culture or smears in the group of participants who had bacteriology confirmed PTB at baseline, i.e. cure rates in intervention group versus control group for participants who initially had sputum AFB-positive, culture-positive or GeneXpert-positive PTB;
- six-month continuous smoking abstinence: the Russell Standard defines continuous abstinence as a self-report of not smoking > 5 cigarettes from the start of the abstinence period (in this case, six months), supported by a negative biochemical test (exhaled carbon monoxide [CO] < 10 ppm) at final follow-up [39]. For the purpose of our study, we will, however, use a more stringent criterion of exhaled CO < 7 ppm, based on findings from our previous study that some participants who reported continued smoking had an exhaled CO < 10 ppm [26]. This analysis will be performed on the group of participants who were current tobacco smokers at baseline;
- reduction in harmful or hazardous drinking: alcohol use will be measured using the AUDIT questionnaire. The questionnaire will be administered at screening (which will take place on the same day or shortly after the baseline assessment) and again at three months and six months. Changes in the total AUDIT score will be used to compare change in drinking behaviour between control and intervention groups. This analysis will be performed on the group of participants who were harmful or hazardous drinkers at baseline.
- TB and ART medication adherence: adherence to both TB and ART medications will be measured using a modified version of the AIDS Clinical Trials Group (ACTG) Adherence Questionnaire. The questionnaire is a validated tool for measuring adherence specifically to ART and we will use an adapted version to also measure TB medication adherence [40]. Field workers will administer the baseline part of this questionnaire as part of the baseline CRF and adherence will be measured at both three months and six months.

Adherence will be measured using an adherence index calculated by the formula (using the four-day recall table):

$$ \left[\mathrm{Total}\ \mathrm{number}\ \mathrm{of}\ \mathrm{doses}\ \mathrm{taken}/\mathrm{Total}\ \mathrm{number}\ \mathrm{of}\ \mathrm{doses}\ \mathrm{prescribed}\right]\ \mathrm{x}\ 100 $$

ART patients with at least 95% of adherence will be considered as having optimal adherence and those with < 95% will be considered as having low (or suboptimal) adherence. For the TB medication regimen, patients with at least 95% of adherence will be considered as having optimal adherence and those with < 95% will be considered as having low (or suboptimal) adherence:

- increase in proportion of HIV-positive participants on ART: HIV status will be recorded in the TB Treatment Record. HIV-positive participants will be asked about ART status at baseline, three months and six months using standardised questions on the CRF.

The following outcomes will be measured at three months:

- biochemically verified three-month sustained tobacco cessation;
- reduction in harmful or hazardous drinking;
- TB drug and ART adherence;
- increase in proportion of HIV-positive participants on ART.

**Fig. 2:**
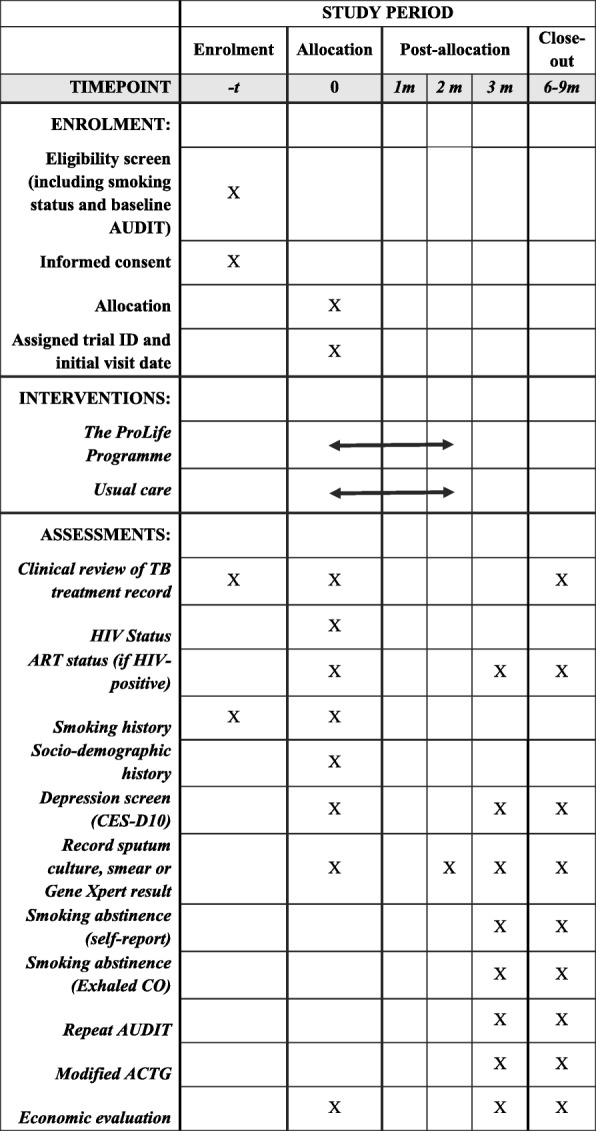
SPIRIT Figure outlining data collection throughout trial

The schedule for enrolment, intervention and assessments is presented in Fig. 2. Table 2 summarises the methods of data collection and analysis for each of the primary and secondary outcomes.

**Table 2:** Summary of methods of data collection and analysis for primary and secondary outcomes

Variable	Method of data collection	Method of data analysis
Primary outcome: TB treatment outcome as defined by WHO and the South Africa TB Management Guidelines (see Table 1)	• CRF and TB treatment record • Treatment outcome as recorded by TB nurses in TB cards using the standard WHO definitions of cure, completions, failure, death, TB treatment interruption, drug resistance developed in course of treatment (confirmed for correctness by checking sputum culture/smear at baseline, at 5 and 6 months)	Binary outcome: successful (cured of completed) vs not successful (died, failure, treatment interruption, drug-resistant TB) All participants, excluding those transferred out
Secondary outcome: sputum conversion	• Sputum culture/smear at baseline, 5 and 6 months to determine cure rates for PTB patients who had a positive smear or culture at baseline • Sputum conversion at 2–3 months	Binary cured vs not cured for the subgroups of participants who had a positive smear/culture at baseline Binary conversion vs not for the subgroups of participants who had a positive smear/culture at baseline
Secondary outcome: change in smoking behaviour	• CRF – questions as per Russell’s Standard – baseline smoking behaviours and then at 3 and 6 months • Exhaled carbon monoxide reading at 3 and 6 months	Binary outcome validated 3 and 6 months sustained smoking cessation for the subgroup of participants who were smoking at baseline
Secondary outcome: percentage reduction in harmful or hazardous drinking (change in AUDIT score)	• AUDIT score at baseline and at 3 and 6 months	Change in AUDIT score (continuous) for subgroups of participants who were hazardous or harmful drinkers at baseline
Secondary outcome: HIV and TB medication adherence	• CRF – questions modified from ACTG	Binary outcome (% adherent vs non-adherent)
Secondary outcome: proportion of HIV-positive participants on ART	• HIV status at baseline • ART status at baseline, 3 months and 6 months	Binary on ART vs not on ART for the subgroup of HIV-positive participants, taking into account baseline % on ART

### Data collection and management

Data will be collected and recorded by field workers equipped with Android phones with a mobile data collection application installed. MI counselling and data collection will take place in a well-ventilated private area inside or outside the clinic (but within the grounds of the health facility); MI sessions will be audio-recorded where consent has been obtained. Fieldworkers and LHWs will be provided with high particulate respirator masks to minimise the risk of infection.

Fieldworkers will follow-up all participants in both arms at three and six months within a window period of two weeks before and two weeks after the ideal three-month and six-month visit. Participants will receive SMS reminders three days before each planned visit. Participants will also be in a position to send ‘please call me’ messages to the district coordinators, who will then call the participant to solve problems that may have arisen with the appointment.

The electronic data captured will be stored on secured and password-protected storage servers and mobile phones which ensure data privacy through only allowing authorised research staff access to the data. The electronic data collection system used for the study requires an SMS gateway to send and receive messages to the research participants. Consenting participants’ phone numbers, participant IDs and associated SMS messages will be stored on the SMS gateway’s secured and password-protected server.

Data quality will be ensured by providing fieldworkers with standard operating procedures (SOPs), training and ongoing support on the importance of data quality, data collection and data collection problem solving. Training will consist of a four-day session before the commencement of the pilot and a one-day training session focusing on problems identified during the pilot preceded the trial. The data manager will continuously monitor the captured data for missing variables and inconsistencies in order to resolve any data problems.

The data manager will export the data from the secured server, conceal the participants study arm allocation and de-identify the data before sharing the data in STATA and R compatible formats. The exported de-identified data will be stored in Dropbox, a secure cloud storage platform, for sharing with the lead trial statistician at the University of York for analysis.

All research data and documents referring to the ProLife trial will be stored and maintained in a secured storage space at SMU for a minimum of 15 years from the end of the ProLife trial. Study materials will be destroyed 15 years after the study.

### Blinding and limitation of risk of bias

This is a complex behavioural intervention and the team dynamics mean that all team members work very closely with one another. As such, LHWs and participants cannot be blinded to the intervention. The determination of the primary outcome will be done by the TB nurses who are blinded to the intervention status of the participants based on routinely collected data. Blinding of the field researchers collecting other questionnaire data (Additional file 2) will not be possible. The statistician will be blinded to the intervention or control arm allocation of participants during the analysis stage.

There is a potential of biased outcome reporting on self-reported tobacco smoking or alcohol consumption favouring the intervention arm. This will be minimised by training the fieldworkers in open communication and standardised data collection. Tobacco cessation will also be verified by exhaled CO monitoring. Given the use of the TB treatment record as a primary source of information pertaining to TB outcomes, there is a risk of non-differential misclassification. However, this risk is equal in both the treatment and control arms and therefore the decision has been taken that the TB treatment record can be used as a primary data source (i.e. bias would be towards the null).

### Sample size

The sample size has been set at 696 participants (348 participants per arm). This sample size is sufficient to detect a 10% difference in TB treatment success rates (0.86 vs 0.76) in the ProLife group versus the control group with 80% power, a significance level of 0.05 and 25% attrition. The sample size per clinic was in the range of 14–74 participants per clinic with a median of 24. The assumed success rates in the control group are based on actual success rates in TB patients in the studied provinces.

### Statistical analysis

We will summarise baseline data descriptively by trial arm; however, we will not undertake statistical comparisons. For continuous measures, we will report means and standard deviations (SD); for skewed data, we will also provide medians and interquartile ranges. For categorical data, we will report counts and percentages. For the primary outcome, we will conduct analysis on an intention-to-treat basis. We will use binary logistic regression to compare the main outcome between the intervention and the usual care arm.

We will carry out similar analyses for the secondary outcomes with appropriate regression techniques: logistic regression for categorical outcomes and linear regression for continuous outcomes. We will also adjust for baseline characteristics and other covariates (HIV status, sex, alcohol versus tobacco versus both, district) if these differ between trial arms at baseline.

In case of missing data, we will employ a number of methods including multiple imputations to assess the sensitivity of the results. We will conduct subgroup analyses to determine whether TB treatment outcomes differ between subgroups, as follows: HIV-positive versus HIV-negative participants; participants with an alcohol problem only versus smokers only versus participants who are conjoint smokers and drinkers; and participants who were GeneXpert positive versus participants who were GeneXpert negative at baseline.

We will follow the CONSORT statement guidelines in reporting. We will use the statistical packages STATA and R to carry out the analyses and a *P* value < 0.05 will be considered as statistically significant. The statistical analysis pertaining to cost-effectiveness will be described in the section detailing the planned economic evaluation.

### Monitoring

Each centre will be responsible for its own data entry and local trial management. Monitoring and site training will be carried out at each site within specified intervals. The project manager will visit each district every third month with the counselling supervisor. The district coordinators will visit each clinic bi-weekly; the site leads and data manager will visit the sites when required.

The monitoring will adhere to the principles of Good Clinical Practice and will follow an agreed monitoring plan. During their bi-weekly visits, the district coordinators will be guided by a checklist to confirm adherence to protocol, review eligibility verification and consent procedures, and provide additional training as needed. Any adverse events will be formally recorded and reported where appropriate.

The mobile data collection tool was improved based on findings from the pilot phase and feedback from the field staff capturing the data. Validation was added to the mobile questionnaires to ensure that all questions should be answered, participants only answer question relevant to them and that no unusual values (range checks) are entered. Coordinators can use the mobile data collection tool on their phones to monitor incomplete data activities, outstanding and upcoming appointments with participants and basic project performance indicators per clinic.

The data captured during the interviews with the participants are only captured on the electronic mobile data collection tool and can therefore not be verified by the site coordinators. However, the district coordinators will require access to all (enrolled TB) patient medical records including, but not limited to, laboratory test results and prescriptions. They will do spot checks by comparing the captured data with the medical records and look for missed outcomes reporting: verify completeness; consistency; and accuracy of data being entered on CRFs. The site leads (or delegated personnel) should work with the district coordinators to ensure that any problems detected are resolved.

The data will also be checked by the data manager for missing or unusual values (range checks) and checked for consistency within participants over time. If any such problems are identified, the individual centres will be contacted and asked to verify or correct the entry.

Indicators related to enrolment, premature withdrawal, motivational interviewing completion, CRF completion and SMS delivery will be analysed and included in monthly reports.

As the ProLife programme is a low-risk intervention, the decision was taken not to appoint an external data monitoring committee.

### Training and intervention fidelity monitoring

Eighteen LHWs, three district coordinators and one person who will be focusing on counselling supervision underwent MI training over five days, which covered: basic knowledge of TB; treatment adherence; tobacco and alcohol use; overview of the overall spirit of MI and communication style; core interviewing skills; evoking change talk, hope and confidence; developing a change plan; and strengthening commitment. The LHWs’ knowledge in terms of TB, alcohol use, tobacco smoking, TB and ART treatment adherence, and motivational interviewing was assessed before and after the training

To ensure that all the essential steps, techniques and skills of an MI session are covered during each session, the LHWs will be provided with a checklist to use during each session (Additional file 3). At the end of each session, they will also complete a post-session semi-structured form onto which they will indicate the extent to which they implemented each element of MI, as well as their general qualitative impressions of that particular session.

During the trial, the LHWs will receive regular supervisory support from the district and national study coordinators. In addition, an appointed supervisor with proficiency in MI will be providing monthly counselling supervision to the LHWs in each site, by travelling to each of the three sites and then providing support to the LHWs in a group, followed by individualised support to each LHW separately. All lay counsellors completed grade 12 and have at least one year of prior counselling experience in TB and/or HIV.

In order to assess the fidelity of the counsellors’ delivery of motivational interviewing during the trial, the validated Motivational Interviewing Treatment Integrity Coding Manual 4.2.1 (MITI) tool will be used [41]. We will tape record each LHW’s counselling sessions and then randomly select 5% of each counsellor’s patients and then use the recordings of all three sessions with those patients for the MITI task. Those selected recordings will be transcribed and translated into English. Two independent raters who are Setswana/Sesotho speakers will listen to the recordings and code a randomly selected 20-min portion of the written transcript. In the case of shorter counselling sessions, the entire recording will be assessed. The coding will entail making ‘Global Ratings’ (on four dimensions: Cultivating change talk; Softening sustain talk; Partnership; and Empathy) and ‘Behaviour Counts’ (with respect to the items: giving information; persuade; persuade with permission; question; simple reflection; complex reflection; affirm; seeking collaboration; emphasising autonomy; and confront). A score will be assigned to each of these items and the scores compared against the competency and proficiency thresholds that are specified in the MITI manual.

### Economic evaluation

The economic evaluation will be a within-trial incremental cost-effectiveness analysis of the ProLife package over and above usual care conducted alongside the RCT. In each arm of the trial, we will estimate the costs of delivering the intervention to participants. Costs consist of the delivery of ProLife intervention (staff costs, overheads and consumables) in the intervention arm and the costs of behavioural support and support (staff time, overheads and consumables) in the control arm. Costs will be calculated based on the length of time of contacts and the unit cost of the healthcare worker delivering the intervention. These costs should demonstrate some within patient variability as cost will be partly dependent upon the length of the recorded appointments.

We will also record contacts with health services for TB by patients in each trial arm, using the questionnaires adapted from those used in a previous trial of TB [42]. Applying unit costs of healthcare to quantities recorded in the service use questionnaire will produce a healthcare cost profile for each patient at baseline, three-month and six-month follow-ups. Healthcare cost profiles based on TB contacts can then be used to investigate differences in total costs between the treatment and control groups. The base case analysis will use healthcare costs as the study perspective. This is justified by presenting the cost implications of delivering the ProLife intervention from a healthcare decision maker’s perspective. However, we will also record participants’ travel costs associated with making the journey to the intervention site. This will estimate the wider societal cost to patients associated with receiving treatment.

We will estimate the cost per additional successful TB outcome by computing an incremental cost-effectiveness ratio (ICER) combining treatment and wider healthcare costs with outcome data from the trial. The analysis will also use measures of health utility. We have registered on the EuroQol website to use the country specific version of EQ-5D-3 L for South Africa to measure health-related quality of life at baseline, three-month and six-month follow-ups. We will use EQ-5D-3 L to estimate changes in quality-adjusted life years (QALY) using the area under the curve by using the utility score at each time point to create a profile [41]. A cost utility analysis is then performed by combining incremental health outcomes using QALYs with the incremental cost. The cost utility analysis will estimate the cost per QALY that estimates the value for money afforded by administering the ProLife intervention over and above usual care. We will plot incremental cost against incremental outcome using a cost-effectiveness plane. We will also conduct sensitivity analyses to assess the robustness of the ICER and use bootstrapping techniques to calculate cost-effectiveness acceptability curves. Supporting analysis will also present the costs to patients incurred by making visits to both trial arms to present the wider cost burden. However, these costs will not be included in the base case scenario as the objective is to present the value for money from the perspective of the purchaser or commissioner.

## Discussion

Ending the TB epidemic is one of the Sustainable Development Goals (SDG) established by the United Nations. The WHO calls for a 90% reduction in TB incidence by 2030 compared to 2015 and has highlighted the need for intensified research and innovation. The WHO has recently published guidance advocating the use of digital technologies, specifically mobile text messaging campaigns, in the treatment of TB as a means of improving the provision of patient-centred care and supporting medication adherence [43]. This publication highlights advances in mobile technologies and network coverage as opportunities to more effectively support patients during what can be a long period of treatment. This study is well-aligned with this guidance and, to our knowledge, this is the first trial looking at a complex behavioural intervention aiming to use MI techniques, alongside SMS, to modify a range of harmful lifestyle factors with the aim of improving TB treatment outcomes.

Work already undertaken in the formative phase suggests that the ProLife intervention is feasible and acceptable to patients. However, we anticipate some potential procedural challenges throughout the trial as we recognise that patients with TB may feel unwell and have taken steps to ensure that the MI and data collection are as brief and comfortable for patients as possible. The district coordinators will call participants to follow-up with those who missed appointments.

Implementing complex interventions in the real world can pose logistical challenges and careful process evaluation will be required to fully understand the challenges that may be presented. However, there is evidence to suggest that using MI techniques can be cost-effective when implemented in healthcare settings and the ProLife programme could represent a scalable and feasible approach to improving the care of patients with TB globally. Its scalability is particularly relevant in South Africa as the government plans to formally introduce community health workers as part of its health workforce.

## Trial status

Protocol Version 1.2, 5 January 2018. Recruitment ongoing; piloting started at 30 clinics: 7 May 2018 and was completed by 31 August. The actual trial enrolment started on 13 November 2018. We expect to enrol the target sample size by May 2019 and plan to continue with follow-up until December 2019. The trial was piloted internally from May 2018 until the end of August 2018; piloting results will be reported at a later stage, together with the trial results.

## Additional files

Additional file 1 Participant consent form for screening and for trial. (DOCX 94 kb)

Additional file 2 Case report form. (DOCX 58 kb)

Additional file 3 Counselling Activities Reporting Form. (DOC 103 kb)

Additional file 4 SPIRIT Checklist. (DOC 120 kb)

## Data Availability

The data sharing plans for the current study are unknown and will be made available at a later date. Research results will be published in international indexed journals and disseminated to managers of the provincial and national Departments of Health. Furthermore, the provincial and national Departments of Health will be involved in the design and final evaluation of the project so as to assure their ownership, sustainability and scaling the programme beyond the life of the trial.

## Abbreviations

ACTG: AIDS Clinical Trials Group
ART: Anti-retroviral therapy
AUDIT: Alcohol Use Disorders Identification Test
CO: Carbon monoxide
CRF: Case report form
EPTB: Extra-pulmonary TB
HIV: Human immunodeficiency virus
LHW: Lay health worker
MI: Motivational interviewing
PTB: Pulmonary TB
SADHS: South Africa Demographic and Health Survey
SMS: Short Message Service
TB: Tuberculosis
