# Supplementary material for: ImPROving TB outcomes by modifying LIFE-style behaviours through a brief motivational intervention followed by short text messages (ProLife): study protocol for a randomised controlled trial
Source: Trials. 2019 Jul 26;20:457. doi: 10.1186/s13063-019-3551-9 (PMC6660690; doi:10.1186/s13063-019-3551-9)
Supplement: Supplementary file 1 — Participant consent form for screening and for trial. (DOCX 94 kb) [file 13063_2019_3551_MOESM1_ESM.docx]

# PATIENT / PARTICIPANT’S INFORMATION & INFORMED CONSENT DOCUMENT FOR THE SCREENING

***(Each patient must receive, read and understand this document before the start of the study)***

**TRIAL TITLE: Im*pro*ving TB outcomes by modifying *life*-style behaviours through a brief motivational intervention (Phase II)**

SPONSOR: Sefako Makgatho University

Principal Investigators: Prof OA Ayo-Yusuf (Sefako Makgatho University/ University of Pretoria), Prof K Siddiqi (University of York)

Institution: Sefako Makgatho University

DAYTIME AND AFTER HOURS TELEPHONE NUMBER(S):

Daytime numbers: 012 521 4611

After hours: 083 442 1970

DATE AND TIME OF FIRST INFORMED CONSENT DISCUSSION:

| Date   \| D \| D \| M \| M \| Y \| Y \| Y \| Y \| \| --- \| --- \| --- \| --- \| --- \| --- \| --- \| --- \| \| H \| H \| M \| M \| Y \| Y \| Y \| Y \| | Time   \| H \| H \| M \| M \| \| --- \| --- \| --- \| --- \| \| H \| H \| M \| M \| |
| --- | --- | --- | --- | --- | --- | --- | --- | --- | --- | --- | --- | --- | --- | --- | --- | --- | --- | --- | --- | --- | --- | --- | --- | --- | --- |

## INTRODUCTION

Good day.

You are invited to participate in a screening survey for a research study. Before you decide whether or not to take part, here is why we are doing the study and what it involves.

As you may know, tuberculosis (TB) is a serious concern in our country. There are many factors that influence successful TB outcomes, including HIV, treatment adherence, smoking and alcohol use. A team of local and international stakeholders and experts will develop an intervention that will target tobacco smoking, problem alcohol drinking and TB and antiretroviral treatment adherence. This study is funded by the Newton Fund and Medical Research Council.

## WHAT IS THE PROLIFE TRIAL ABOUT? WHY ARE YOU ASKED TO PARTICIPATE IN THIS STUDY?

The Prolife study is a research study on tobacco smoking, alcohol and treatment adherence in TB patients and we are inviting you to take part in this study. The study is funded by the Medical Research Council-Newton Foundation and is approved by the ethics committees of Sefako Makgatho University, the University of York, the University of Pretoria, the University of Witwatersrand and South African Medical Research Council in South Africa. Here, we explain why we are doing the Prolife Study and what it will involve. This will help you to decide whether to participate in this study. You are free to choose whether or not to participate in this study.

The purpose of this screening procedure is to collect information about you to determine whether or not you qualify to take part in the study. This screening will involve you answering some questions on your current health, smoking and alcohol drinking behaviour. A research assistant will also examine your medical records during this screening procedure. In order to qualify, you must be an adult with pulmonary tuberculosis who smokes tobacco or who drinks alcohol.

## WHAT WILL I HAVE TO DO?

You will have to give consent for the screening including the examining of your medical records. If you agree to the screening procedure (or to participate) you will be asked a series of questions to determine your eligibility. These questions include information about your current health, treatment and smoking and drinking behaviour. This screening procedure will take approximately 10 minutes.

## ARE THERE ANY RISKS OR DISCOMFORT INVOLVED?

There are no direct risks to your health but you might feel uncomfortable in answering certain questions. If you do, you may withdraw from the screening at any time.

## IF I TAKE PART WILL MY INFORMATION BE KEPT CONFIDENTIAL?

All the information we collect from you on the paper documents will be kept confidential on secure computers and in locked filing cabinets in locked offices and transferred safely to the country’s coordinating centre at Sefako Makgatho University to be secured.

Data that will be collected using the mobile phone, including your phone number, will be securely stored on the mobile phone and an online storage space. Only authorized researchers and personnel will be able to access the information. After we are finished collecting data, your data will be de-identified (all information that might be traced to you will be removed) and then it will be shared with the researchers working on this project.

The research staff will ensure that your identity and location in this study will remain strictly confidential. However, there is a slight risk that your information and number might get stolen by malicious people and used inappropriately.

Make sure that you provide your own phone numbers or the number of a person who you feel comfortable with to share information about TB, alcohol, and tobacco.

The results of the study, including data, may be published for scientific purposes but will not give your name or include any identifiable references to you.

The study materials will be kept for 15 years; after which time they will be destroyed.

## WHAT ARE THE POSSIBLE BENEFITS OF TAKING PART IN THE STUDY?

You may qualify to participate in the research study and it is hoped that the information collected from the study will help the researches to develop an intervention which may benefit your health.

## REIMBURSEMENT

You will not receive reimbursement for the screening. You will also not be charged for the screening.

## HAS THE STUDY RECEIVED ETHICAL APPROVAL?

This Protocol was submitted to the Research Ethics Committees of the relevant Universities: Sefako Makgatho University; University of York; University of Witwatersrand; the University of Pretoria, the Medical Research Council; and the University of the Free State. The study has been structured in accordance with the Declaration of Helsinki (last update: October 2013), which deals with the recommendations guiding doctors in biomedical research involving human/subjects. A copy of the Declaration may be obtained from the investigator should you wish to review it. If you have any queries related to ethics, you may contact:

## WHERE CAN I OBTAIN ADDITIONAL INFORMATION?

If I have any questions concerning this study, I should contact:

- M Malefo, cell: 072 6412747
- Prof Olu Omole, tel: 016 950 6192 cell: 076 472 1289 (Sedibeng district)
- Prof John Tumbo, tel: 012 521 4314 or cell: 082 885 8332 (Bojanala district)
- Dr Michelle Engelbrecht, tel: 051 401 3256 or cell: 083 304 8182 (Lejweleputswa district)

If I have any queries related to ethics, I should contact:

- Dr C.Baker at Sefako Makgatho Health Sciences University (telephone number: 012 521 5617);
- Dr S Holland at the University of York (telephone number: 019 0432 4031);
- University of the Witwatersrand
  - Prof P Cleaton Jones, Chair, Tel 011 717 2301, Email: peter.cleaton-jones1@wits.ac.za
  - Ms Z Ndlovu, Administrative Officer, Email: zanele.ndlovu@wits.ac.za
  - Mr Rhulani Mkansi, Administrative Officer, Email: rhulani.mkansi@wits.ac.za
  - Mr Lebo Moeng, Administrative Officer, Email: lebo.moeng@wits.ac.za
  - Tel: 011 717 2700/2656/1234/1252
- 4. Professor W Van Staden at the University of Pretoria (telephone numbers: 012 354 1677/ 012 354 1330);
- Professor D du Toit at the South African Medical Research Council (telephone number: 021 938 0687).
- 6. The Chairperson: University of the Free State Ethics Committee: Health Sciences

Block D, Deans Division, Room D104

PO Box 2339 (Internal Box G40)

Bloemfontein

Tel: 051 – 401 7795

e-mail: EthicsFHS@ufs.ac.za

## CONSENT TO PARTICIPATE IN THIS STUDY

I have read or had read to me in a language that I understand the above information before signing this consent form. The content and meaning of this information have been explained to me. I have been given opportunity to ask questions and am satisfied that they have been answered satisfactorily. I understand that if I do not participate it will not alter my management in any way. I hereby volunteer to take part in this screening stage of the study.

***I have received a signed copy of this informed consent agreement.***

| (The study participant’s name and date must be completed by the investigator) | |
| --- | --- |
| Study participant’s Name   \|  \|  \|  \|  \|  \|  \|  \|  \|  \|  \|  \|  \|  \|  \|  \|  \|  \|  \|  \|  \|  \| \| --- \| --- \| --- \| --- \| --- \| --- \| --- \| --- \| --- \| --- \| --- \| --- \| --- \| --- \| --- \| --- \| --- \| --- \| --- \| --- \| --- \| | |
| Study participant’s Surname   \|  \|  \|  \|  \|  \|  \|  \|  \|  \|  \|  \|  \|  \|  \|  \|  \|  \|  \|  \|  \|  \| \| --- \| --- \| --- \| --- \| --- \| --- \| --- \| --- \| --- \| --- \| --- \| --- \| --- \| --- \| --- \| --- \| --- \| --- \| --- \| --- \| --- \| | |
| Date   \| D \| D \| M \| M \| Y \| Y \| Y \| \| --- \| --- \| --- \| --- \| --- \| --- \| --- \| | Study participant’s Signature   \|  \| \| --- \| |
| Investigator's Name   \|  \|  \|  \|  \|  \|  \|  \|  \|  \|  \|  \|  \|  \|  \|  \|  \|  \|  \|  \|  \|  \| \| --- \| --- \| --- \| --- \| --- \| --- \| --- \| --- \| --- \| --- \| --- \| --- \| --- \| --- \| --- \| --- \| --- \| --- \| --- \| --- \| --- \| | |
| Investigator's Surname   \|  \|  \|  \|  \|  \|  \|  \|  \|  \|  \|  \|  \|  \|  \|  \|  \|  \|  \|  \|  \|  \| \| --- \| --- \| --- \| --- \| --- \| --- \| --- \| --- \| --- \| --- \| --- \| --- \| --- \| --- \| --- \| --- \| --- \| --- \| --- \| --- \| --- \| | |
| Date   \| D \| D \| M \| M \| Y \| Y \| Y \| \| --- \| --- \| --- \| --- \| --- \| --- \| --- \| | Investigator's Signature   \|  \| \| --- \| |
| Witness's Name Name   \|  \|  \|  \|  \|  \|  \|  \|  \|  \|  \|  \|  \|  \|  \|  \|  \|  \|  \|  \|  \|  \| \| --- \| --- \| --- \| --- \| --- \| --- \| --- \| --- \| --- \| --- \| --- \| --- \| --- \| --- \| --- \| --- \| --- \| --- \| --- \| --- \| --- \| | |
| Witness's Name Surname   \|  \|  \|  \|  \|  \|  \|  \|  \|  \|  \|  \|  \|  \|  \|  \|  \|  \|  \|  \|  \|  \| \| --- \| --- \| --- \| --- \| --- \| --- \| --- \| --- \| --- \| --- \| --- \| --- \| --- \| --- \| --- \| --- \| --- \| --- \| --- \| --- \| --- \| | |
| Date   \| D \| D \| M \| M \| Y \| Y \| Y \| \| --- \| --- \| --- \| --- \| --- \| --- \| --- \| | Witness's Signature   \|  \| \| --- \| |

## VERBAL PATIENT INFORMED CONSENT

(This section is only applicable to participants who are unable to read and write)

I, the undersigned, _________________________, have read and have explained fully to the study participant’s, named _________________and/or his/her relative, the patient information leaflet, which has indicated the nature and purpose of the study in which I have asked the patient to participate. The explanation I have given has mentioned both the possible risks and benefits of the study and the alternative treatments available for his/her illness. The patient indicated that he/she understands that he/she will be free to withdraw from the study at any time for any reason and without jeopardizing his/her treatment.

The patient consents to the obtaining of information from his/her TB file to determine the type of TB she/he has and to determine for how long she/he has been on treatment.

***I hereby certify that the patient has agreed to participate in this study.***

| (The study participant’s name and date must be completed by the investigator) | |
| --- | --- |
| Study participant’s Name   \|  \|  \|  \|  \|  \|  \|  \|  \|  \|  \|  \|  \|  \|  \|  \|  \|  \|  \|  \|  \|  \| \| --- \| --- \| --- \| --- \| --- \| --- \| --- \| --- \| --- \| --- \| --- \| --- \| --- \| --- \| --- \| --- \| --- \| --- \| --- \| --- \| --- \| | |
| Study participant’s Surname   \|  \|  \|  \|  \|  \|  \|  \|  \|  \|  \|  \|  \|  \|  \|  \|  \|  \|  \|  \|  \|  \| \| --- \| --- \| --- \| --- \| --- \| --- \| --- \| --- \| --- \| --- \| --- \| --- \| --- \| --- \| --- \| --- \| --- \| --- \| --- \| --- \| --- \| | |
| Date   \| D \| D \| M \| M \| Y \| Y \| Y \| \| --- \| --- \| --- \| --- \| --- \| --- \| --- \| | Study participant’s Mark/Thumb print   \|  \| \| --- \| |
| Investigator's Name   \|  \|  \|  \|  \|  \|  \|  \|  \|  \|  \|  \|  \|  \|  \|  \|  \|  \|  \|  \|  \|  \| \| --- \| --- \| --- \| --- \| --- \| --- \| --- \| --- \| --- \| --- \| --- \| --- \| --- \| --- \| --- \| --- \| --- \| --- \| --- \| --- \| --- \| | |
| Investigator's Surname   \|  \|  \|  \|  \|  \|  \|  \|  \|  \|  \|  \|  \|  \|  \|  \|  \|  \|  \|  \|  \|  \| \| --- \| --- \| --- \| --- \| --- \| --- \| --- \| --- \| --- \| --- \| --- \| --- \| --- \| --- \| --- \| --- \| --- \| --- \| --- \| --- \| --- \| | |
| Date   \| D \| D \| M \| M \| Y \| Y \| Y \| \| --- \| --- \| --- \| --- \| --- \| --- \| --- \| | Investigator's Signature   \|  \| \| --- \| |
| Witness's Name Name   \|  \|  \|  \|  \|  \|  \|  \|  \|  \|  \|  \|  \|  \|  \|  \|  \|  \|  \|  \|  \|  \| \| --- \| --- \| --- \| --- \| --- \| --- \| --- \| --- \| --- \| --- \| --- \| --- \| --- \| --- \| --- \| --- \| --- \| --- \| --- \| --- \| --- \| | |
| Witness's Name Surname   \|  \|  \|  \|  \|  \|  \|  \|  \|  \|  \|  \|  \|  \|  \|  \|  \|  \|  \|  \|  \|  \| \| --- \| --- \| --- \| --- \| --- \| --- \| --- \| --- \| --- \| --- \| --- \| --- \| --- \| --- \| --- \| --- \| --- \| --- \| --- \| --- \| --- \| | |
| Date   \| D \| D \| M \| M \| Y \| Y \| Y \| \| --- \| --- \| --- \| --- \| --- \| --- \| --- \| | Witness's Signature   \|  \| \| --- \| |

(Witness signs that he/she has witnessed the process of informed consent)

## CONSENT TO EXAMINE PATIENT’S MEDICAL RECORD

I have read or had read to me in a language that I understand the information about this study before signing this consent form. The content and meaning of this information have been explained to me. I have been given opportunity to ask questions and I am satisfied that they have been answered satisfactorily. I understand that if I do not give consent for my medical records to be examined by the researchers, it will not alter my management in any way. I hereby consent to my medical records being examined at this stage of the study.

***I have received a signed copy of this informed consent agreement.***

| (The study participant’s name and date must be completed by the investigator) | |
| --- | --- |
| Study participant’s Name   \|  \|  \|  \|  \|  \|  \|  \|  \|  \|  \|  \|  \|  \|  \|  \|  \|  \|  \|  \|  \|  \| \| --- \| --- \| --- \| --- \| --- \| --- \| --- \| --- \| --- \| --- \| --- \| --- \| --- \| --- \| --- \| --- \| --- \| --- \| --- \| --- \| --- \| | |
| Study participant’s Surname   \|  \|  \|  \|  \|  \|  \|  \|  \|  \|  \|  \|  \|  \|  \|  \|  \|  \|  \|  \|  \|  \| \| --- \| --- \| --- \| --- \| --- \| --- \| --- \| --- \| --- \| --- \| --- \| --- \| --- \| --- \| --- \| --- \| --- \| --- \| --- \| --- \| --- \| | |
| Date   \| D \| D \| M \| M \| Y \| Y \| Y \| \| --- \| --- \| --- \| --- \| --- \| --- \| --- \| | Study participant’s Signature   \|  \| \| --- \| |
| Investigator's Name   \|  \|  \|  \|  \|  \|  \|  \|  \|  \|  \|  \|  \|  \|  \|  \|  \|  \|  \|  \|  \|  \| \| --- \| --- \| --- \| --- \| --- \| --- \| --- \| --- \| --- \| --- \| --- \| --- \| --- \| --- \| --- \| --- \| --- \| --- \| --- \| --- \| --- \| | |
| Investigator's Surname   \|  \|  \|  \|  \|  \|  \|  \|  \|  \|  \|  \|  \|  \|  \|  \|  \|  \|  \|  \|  \|  \| \| --- \| --- \| --- \| --- \| --- \| --- \| --- \| --- \| --- \| --- \| --- \| --- \| --- \| --- \| --- \| --- \| --- \| --- \| --- \| --- \| --- \| | |
| Date   \| D \| D \| M \| M \| Y \| Y \| Y \| \| --- \| --- \| --- \| --- \| --- \| --- \| --- \| | Investigator's Signature   \|  \| \| --- \| |
| Witness's Name Name   \|  \|  \|  \|  \|  \|  \|  \|  \|  \|  \|  \|  \|  \|  \|  \|  \|  \|  \|  \|  \|  \| \| --- \| --- \| --- \| --- \| --- \| --- \| --- \| --- \| --- \| --- \| --- \| --- \| --- \| --- \| --- \| --- \| --- \| --- \| --- \| --- \| --- \| | |
| Witness's Name Surname   \|  \|  \|  \|  \|  \|  \|  \|  \|  \|  \|  \|  \|  \|  \|  \|  \|  \|  \|  \|  \|  \| \| --- \| --- \| --- \| --- \| --- \| --- \| --- \| --- \| --- \| --- \| --- \| --- \| --- \| --- \| --- \| --- \| --- \| --- \| --- \| --- \| --- \| | |
| Date   \| D \| D \| M \| M \| Y \| Y \| Y \| \| --- \| --- \| --- \| --- \| --- \| --- \| --- \| | Witness's Signature   \|  \| \| --- \| |

## VERBAL CONSENT TO EXAMINE MEDICAL RECORDS

**(This section is only applicable to participants who are unable to read and write)**

I, the undersigned, _________________________, have read and have explained fully to the study participant’s, named _________________and/or his/her relative, the patient information leaflet, which has indicated the nature and purpose of the study in which I have asked the patient to consent that his/her medical record be examined. The patient indicated that he/she understands that he/she will be free to withdraw her/his consent at any time, for any reason and without jeopardizing his/her treatment.

The patient consents to the obtaining of information from his/her TB file to determine the type of TB she/he has and to determine for how long she/he has been on treatment.

***I hereby certify that the patient has agreed to participate in this study.***

| (The study participant’s name and date must be completed by the investigator) | |
| --- | --- |
| Study participant’s Name   \|  \|  \|  \|  \|  \|  \|  \|  \|  \|  \|  \|  \|  \|  \|  \|  \|  \|  \|  \|  \|  \| \| --- \| --- \| --- \| --- \| --- \| --- \| --- \| --- \| --- \| --- \| --- \| --- \| --- \| --- \| --- \| --- \| --- \| --- \| --- \| --- \| --- \| | |
| Study participant’s Surname   \|  \|  \|  \|  \|  \|  \|  \|  \|  \|  \|  \|  \|  \|  \|  \|  \|  \|  \|  \|  \|  \| \| --- \| --- \| --- \| --- \| --- \| --- \| --- \| --- \| --- \| --- \| --- \| --- \| --- \| --- \| --- \| --- \| --- \| --- \| --- \| --- \| --- \| | |
| Date   \| D \| D \| M \| M \| Y \| Y \| Y \| \| --- \| --- \| --- \| --- \| --- \| --- \| --- \| | Study participant’s Mark/Thumbprint   \|  \| \| --- \| |
| Investigator's Name   \|  \|  \|  \|  \|  \|  \|  \|  \|  \|  \|  \|  \|  \|  \|  \|  \|  \|  \|  \|  \|  \| \| --- \| --- \| --- \| --- \| --- \| --- \| --- \| --- \| --- \| --- \| --- \| --- \| --- \| --- \| --- \| --- \| --- \| --- \| --- \| --- \| --- \| | |
| Investigator's Surname   \|  \|  \|  \|  \|  \|  \|  \|  \|  \|  \|  \|  \|  \|  \|  \|  \|  \|  \|  \|  \|  \| \| --- \| --- \| --- \| --- \| --- \| --- \| --- \| --- \| --- \| --- \| --- \| --- \| --- \| --- \| --- \| --- \| --- \| --- \| --- \| --- \| --- \| | |
| Date   \| D \| D \| M \| M \| Y \| Y \| Y \| \| --- \| --- \| --- \| --- \| --- \| --- \| --- \| | Investigator's Signature   \|  \| \| --- \| |
| Witness's Name Name   \|  \|  \|  \|  \|  \|  \|  \|  \|  \|  \|  \|  \|  \|  \|  \|  \|  \|  \|  \|  \|  \| \| --- \| --- \| --- \| --- \| --- \| --- \| --- \| --- \| --- \| --- \| --- \| --- \| --- \| --- \| --- \| --- \| --- \| --- \| --- \| --- \| --- \| | |
| Witness's Name Surname   \|  \|  \|  \|  \|  \|  \|  \|  \|  \|  \|  \|  \|  \|  \|  \|  \|  \|  \|  \|  \|  \| \| --- \| --- \| --- \| --- \| --- \| --- \| --- \| --- \| --- \| --- \| --- \| --- \| --- \| --- \| --- \| --- \| --- \| --- \| --- \| --- \| --- \| | |
| Date   \| D \| D \| M \| M \| Y \| Y \| Y \| \| --- \| --- \| --- \| --- \| --- \| --- \| --- \| | Witness's Signature   \|  \| \| --- \| |

*(Witness signs that he/she has witnessed the process of informed consent)*

# SCREENING TOOL

***Please indicate Yes (Y) or No (N) to the following questions***

|  | | No | Yes |
| --- | --- | --- | --- |
| 1. Is the patient 18 years or older? (**If “No” please end the interview**). | | 0 | 1 |
| 1. Does patient have pulmonary TB? (To be extracted from the TB blue card or from information from the TB nurse on admission to the clinic).   (**If “No” please end the interview**). | | 0 | 1 |
| 1. Has the patient been on treatment for more than four weeks? (To be extracted from the TB blue card or the green card or the pink hospital referral form or from information from the TB nurse on admission to the clinic). (**If “Yes” please end the interview).** | | 0 | 1 |
| 1. Do you speak and understand any of the following languages: Zulu or Setswana or Sesotho or English? | | 0 | 1 |
| 1. Do you have easy access to a functional cell phone? This will allow us to SMS you supportive messages and send you reminders regarding appointments.   If yes, what is your cell phone number: _________________  Alternative cell phone number: _________________  **If No easily accessible cell phone, please end the interview** | | 0 | 1 |
| 1. Name of the clinic or health centre: |  | | |
| 1. What is your age? | _______________________ (Age in completed years) | | |
|  | | Male | Female |
| 1. Sex | | 0 | 1 |
|  | |  |  |
| 9. In the past month, have you smoked tobacco on a daily basis, less than daily basis or not at all?. (*This question refers to smoking cigarettes, cigars, pipes, cigarillos and Hubbly Bubbly/waterpipe but excludes Smokeless tobacco use.)*   - Daily [1] - Less than Daily [2] - Not at all [0]   [If 1 or 2, go to “Smoking Section” of the CRF, If 0 go to the “Smokeless tobacco” section if eligible] | |  |  |
|  | | No | Yes |
| 10. Have you had a drink containing alcohol in the past 12 months? | | 0 | 1 |

**[If ZERO for question 9 and NO for questions 10 interview will end. Otherwise questionnaire will automatically be continued]**

Below is a list of questions about your drinking behaviour. Please circle the score (1-4) that best reflects your behaviour.

| **In the past 12 months**: | **Never** | | **Monthly or less** | | **2 to 4 times a month** | **2 to 3 times a week** | **4 or more times a week** |  | **Sub totals** | |
| --- | --- | --- | --- | --- | --- | --- | --- | --- | --- | --- |
| 1. How often do you have a drink containing alcohol? | 0 | | 1 | | 2 | 3 | 4 |  |  | |
|  | 1 or 2 | | 3 or 4 | | 5 or 6 | 7 to 9 | 10 or more |  |  | |
| 1. How many drinks containing alcohol do you have on a typical day when you are drinking? (Please note that one drink is equivalent to one can or bottle of beer, cider or cooler, one glass of wine, or one tot of spirits). | 0 | | 1 | | 2 | 3 | 4 |  |  | |
|  | **Never** | | **Less than monthly** | | **Monthly** | **Weekly** | **Daily or almost daily** |  |  | |
| 1. How often do you have six or more drinks on one occasion? | 0 | | 1 | | 2 | 3 | 4 |  |  | |
| 1. How often during the last year have you found that you were not able to stop drinking once you have started? | 0 | | 1 | | 2 | 3 | 4 |  |  | |
| 1. How often during the last year have you failed to do what was normally expected from you because of drinking? | 0 | | 1 | | 2 | 3 | 4 |  |  | |
| 1. How often during the last year have you needed a first drink in the morning to get yourself going after a heavy drinking session? | 0 | | 1 | | 2 | 3 | 4 |  |  | |
| 1. How often during the last year have you had a feeling of guilt or remorse after drinking? | 0 | | 1 | | 2 | 3 | 4 |  |  | |
| 1. How often during the last year have you been unable to remember what happened the night before because you had been drinking? | 0 | | 1 | | 2 | 3 | 4 |  |  | |
|  | **No** | | **Yes, but not in the last year** | | | | **Yes, during the last year** |  |  | |
| 1. Have you or someone else been injured as a result of your drinking? | 0 | | 2 | | | | 4 |  |  | |
| 1. Has a relative, friend, or a doctor or another health worker been concerned about your drinking or suggested you cut down? | 0 | | 2 | | | | 4 |  |  | |
|  |  |  | |  | | | |  |  | |
| **Total AUDIT score will be calculated automatically**: | | | | | | | | |  |  |

| Respondent is a CURRENT smoker (smoked tobacco in the past month) | **Eligible**: proceed with consent procedure for RCT |
| --- | --- |
| AUDIT score is ≥8 for men or ≥7 for women AND the AUDIT score is <20 | **Eligible:** proceed with consent procedure for RCT |
| AUDIT score is <8 for men or <7 for women AND the respondent is NOT a current smoker (smoked zero cigarettes/cigars/cigarillos/pipes in the past month) | **Not eligible:** proceed with consent procedure for RCT |
| AUDIT score is ≥20. | **Not eligible:** proceed with consent procedure for RCT |

Thank you very much.

# PATIENT / PARTICIPANT’S INFORMATION & INFORMED CONSENT DOCUMENT FOR THE RANDOMISED CONTROLLED TRIAL

***(Each patient must receive, read and understand this document before the start of the study)***

TRIAL TITLE: Im*pro*ving TB outcomes by modifying *life*-style behaviours through a brief motivational intervention (Phase II)

SPONSOR: Sefako Makgatho University

Principal Investigators: Prof OA Ayo-Yusuf (Sefako Makgatho University/ University of Pretoria), Prof K Siddiqi (University of York)

Institution: Sefako Makgatho University

DAYTIME AND AFTER HOURS TELEPHONE NUMBER(S):

Daytime numbers: 012 521 4611

After hours: 083 442 1970

DATE AND TIME OF FIRST INFORMED CONSENT DISCUSSION:

| Date   \| D \| D \| M \| M \| Y \| Y \| Y \| Y \| \| --- \| --- \| --- \| --- \| --- \| --- \| --- \| --- \| \| H \| H \| M \| M \| Y \| Y \| Y \| Y \| | Time   \| H \| H \| M \| M \| \| --- \| --- \| --- \| --- \| \| H \| H \| M \| M \| |
| --- | --- | --- | --- | --- | --- | --- | --- | --- | --- | --- | --- | --- | --- | --- | --- | --- | --- | --- | --- | --- | --- | --- | --- | --- | --- |

**Dear Patient**

## INTRODUCTION

You have been found to be eligible and, as such, are invited to volunteer for this research study. This information leaflet is to help you to decide if you would like to participate. Before you agree to take part in this study you should fully understand what is involved. If you have any questions, which are not fully explained in this leaflet, do not hesitate to ask the investigator. You should not agree to take part unless you are completely happy about all the procedures involved. In the best interests of your health, it is strongly recommended that you discuss with or inform your personal doctor of your possible participation in this study, wherever possible.

## WHAT IS THE PURPOSE OF THE RESEARCH TRIAL?

As you may know, tuberculosis (TB) is a serious concern in our country. There are many factors that influence successful TB outcomes, including HIV, treatment adherence, smoking and alcohol use. A team of local and international stakeholders and experts developed an intervention that will target tobacco smoking, problem alcohol drinking and TB and antiretroviral treatment adherence.

## WHAT IS THE PROLIFE TRIAL ABOUT? WHY ARE YOU ASKED TO PARTICIPATE IN THIS STUDY?

The Prolife study is a research study on tobacco smoking, alcohol and treatment adherence in TB patients. The study is funded by the Medical Research Council-Newton Foundation and was approved by the ethics committees of Sefako Makgatho University, the University of York, the University of Pretoria, the University of Witwatersrand and South African Medical Research Council in South Africa. Here, we explain why we are doing the Prolife Study and what it will involve. This will help you to decide whether to participate in this study. You are free to choose whether or not to participate in this study.

All participants in this trial will receive the usual treatment for TB and will be seen by the nurse as normal. Around half of the participants will receive some additional sessions (the Prolife package) as well as this. This is decided at random and you will not be able to request to receive this. It is important to realise that no participant will receive anything less than the usual TB treatment as advised by all recognised guidelines.

Those participants who are given the Prolife package will receive three extra sessions one month apart with a lay health worker. These sessions will focus on looking at ways of motivating you to reduce your smoking, reduce your alcohol and to take your medications properly. You will also receive text messages to your phone to reinforce these sessions. Participants who are given the Prolife package will therefore be asked to provide their phone numbers. The sessions with the lay counselors may be recorded. This will be done strictly to study whether the counselor how well the counselor manages the counseling sessions. Your personal information discussed will be kept strictly confidential.

All participants will have 3 sessions with a researcher who will ask you questions about your background, your general health, your smoking and alcohol habits and how well you take your medications. Each session is expected not to last more than 25 minutes. By consenting to take part in this study you are also consenting to allow this researcher access to your TB treatment record. This information will be kept anonymous and confidential by the research team.

## WHAT IS THE DURATION OF THIS TRIAL?

If you decide to take part you will be one of approximately 720 patients. The study will last for up to 6 months. You will be asked to visit the investigator 3 times as per the following schedule: at the beginning, at 3 months and at 6 months.

## I UNDERSTAND THAT IF I DO NOT WANT TO PARTICIPATE IN THIS STUDY, I WILL STILL RECEIVE STANDARD TREATMENT FOR MY ILLNESS.

## I MAY AT ANY TIME WITHDRAW FROM THIS STUDY.

## HAS THE TRIAL RECEIVED ETHICAL APPROVAL?

This Protocol was submitted to the Research Ethics Committees of the relevant Universities: Sefako Makgatho University; University of York; University of Witwatersrand; the University of Pretoria, the Medical Research Council; and the University of the Free State. The study has been structured in accordance with the Declaration of Helsinki (last update: October 2013), which deals with the recommendations guiding doctors in biomedical research involving human/subjects. A copy of the Declaration may be obtained from the investigator should you wish to review it. If you have any queries related to ethics, you may contact:

## WHERE CAN I OBTAIN ADDITIONAL INFORMATION?

If I have any questions concerning this study, I should contact

- Mrs M Malefo, tel: 012 521 4611 or cell: 0726472741
- Prof Olu Omole, tel: 016 950 6192 cell: 076 472 1289 (Sedibeng district
- Prof John Tumbo, tel: 012 521 4314 or cell: 082 885 8332 (Bojanala district
- Dr Michelle Engelbrecht, tel: 051 401 3256 or cell: 083 304 8182 (Lejweleputswa district)

If I have any queries related to ethics, I should contact:

- Dr C.Baker at Sefako Makgatho Health Sciences University (telephone number: 012 521 5617);
- Dr S Holland at the University of York (telephone number: 019 0432 4031);
- University of the Witwatersrand
  - Prof P Cleaton Jones, Chair, Tel 011 717 2301, Email: peter.cleaton-jones1@wits.ac.za
  - Ms Z Ndlovu, Administrative Officer, Email: zanele.ndlovu@wits.ac.za
  - Mr Rhulani Mkansi, Administrative Officer, Email: rhulani.mkansi@wits.ac.za
  - Mr Lebo Moeng, Administrative Officer, Email: lebo.moeng@wits.ac.za
  - Tel: 011 717 2700/2656/1234/1252
- Professor W Van Staden at the University of Pretoria (telephone numbers: 012 354 1677/ 012 354 1330);
- Professor D du Toit at the South African Medical Research Council (telephone number: 021 938 0687).
- The Chairperson: University of the Free State Ethics Committee: Health Sciences

Block D, Deans Division, Room D104

PO Box 2339 (Internal Box G40)

Bloemfontein

Tel: 051 – 401 7795

e-mail: EthicsFHS@ufs.ac.za

## IF I TAKE PART WILL MY INFORMATION BE KEPT CONFIDENTIAL?

All the information we collect from you on the paper documents and during audio recordings will be kept confidential on secure computers and in locked filing cabinets in locked offices and transferred safely to the country’s coordinating centre at Sefako Makgatho University to be secured.

Data that will be collected using the mobile phone, including your phone number, will be securely stored on the mobile phone and an online storage space. Only authorized researchers and personnel will be able to access the information. After we are finished collecting data, your data will be de-identified (all information that might be traced to you will be removed) and then it will be shared with the researchers working on this project.

Your phone number will also be stored on a short text message (SMS) company’s secured sever. This company will assist us to send you appointment reminders and messages about TB, alcohol, and tobacco. The companies and the research team are not allowed to use your number for any other purposes.

The research staff will ensure that your identity and location in this study will remain strictly confidential. However, there is a slight risk that your information and number might get stolen by malicious people and used inappropriately.

Make sure that you provide your own phone numbers or the number of a person who you feel comfortable with to share information about TB, alcohol, and tobacco.

The results of the study, including data, may be published for scientific purposes but will not give your name or include any identifiable references to you.

The study materials will be kept for 15 years; after which time they will be destroyed.

## WHAT ARE YOUR RIGHTS AS A PARTICIPANT IN THIS TRIAL?

Your participation in this trial is entirely voluntary and you can refuse to participate or stop at any time without stating any reason. Your withdrawal will not affect your access to other medical care. The investigator retains the right to withdraw you from the study if it is considered to be in your best interest. If it is detected that you did not give an accurate history or did nor follow the guidelines of the trial and the regulations of the trial facility, you may be withdrawn from the trial at any time.

## IS ALTERNATIVE TREATMENT AVAILABLE?

Alternative treatment in the form of seeing the nurse and having the usual TB treatment given to all patients is available and will help to treat your TB. If you decide not to take part in this study you will still receive all of this usual treatment.

## WHAT ARE THE RISKS INVOLVED IN THIS TRIAL? MAY ANY OF THESE TRIAL PROCEDURES RESULT IN DISCOMFORT OR INCONVENIENCE.

There are no technical procedures involved. However, the follow-up sessions will take some of your time (about 45 minutes of your time: 25 minutes for questions about your background, your general health, your smoking and alcohol habits and how well you take your medications. Another 20 minutes for counselling, if you are allocated to receive counselling from the Lay health worker. This does not include time involved to travel to and from the venue).

## WHAT ARE THE BENEFITS TO YOU?

We hope that we are able to help you in three main ways (one or all of these may apply to you): we hope we can help you to reduce the amount of tobacco you are smoking, the amount of alcohol you consume and, if appropriate, we hope that we can help you to ensure that you improve how you take your TB and/or HIV medication. We think that doing one or all of these might help to improve your recovery from TB and if this is found to be the case, the Prolife package may be used to help many patients with TB in the future.

## REIMBURSEMENT

To cover your travel expenses for the extra visits, you will be reimbursed to the value of 60 Rand per follow-up session.

# CONSENT FOR AUDIO-RECORDING OF COUNSELLING SESSION WITH LAY HEALTH WORKER

***(Each eligible patient must receive, read and understand this document before the start of the counselling)***

TRIAL TITLE: Im*pro*ving TB outcomes by modifying *life*-style behaviours through a brief motivational intervention (Phase II)

SPONSOR: Sefako Makgatho University

Principal Investigators: Prof OA Ayo-Yusuf (Sefako Makgatho University/ University of Pretoria), Prof K Siddiqi (University of York)

Institution: Sefako Makgatho University

DAYTIME AND AFTER HOURS TELEPHONE NUMBER(S):

Daytime numbers: 012 521 4611

After hours: 083 442 1970

DATE AND TIME OF FIRST INFORMED CONSENT DISCUSSION:

| Date   \| D \| D \| M \| M \| Y \| Y \| Y \| Y \| \| --- \| --- \| --- \| --- \| --- \| --- \| --- \| --- \| \| H \| H \| M \| M \| Y \| Y \| Y \| Y \| | Time   \| H \| H \| M \| M \| \| --- \| --- \| --- \| --- \| \| H \| H \| M \| M \| |
| --- | --- | --- | --- | --- | --- | --- | --- | --- | --- | --- | --- | --- | --- | --- | --- | --- | --- | --- | --- | --- | --- | --- | --- | --- | --- |

## CONSENT TO ALLOW AUDIO RECORDING OF COUNSELLING SESSIONS BY LAY HEALTHCARE WORKER

I have read or had read to me in a language that I understand, the above information regarding this trial and the need for an audio recording of the interview before signing this audio-recording consent form. The content and meaning of this information have been explained to me. I have been given opportunity to ask questions and I am satisfied that they have been answered satisfactorily.

I understand that I do not have to give consent to this audio recording. I further understand that I can request that the tape recorder be turned off at any time and may request that the tape or any portion thereof be erased.

I may terminate this permission to audio-tape at any time. The contents of these taped sessions are confidential and the information will not be shared outside the context of this research. The tapes will be stored in a secure location and will not be used for any other purpose without my explicit written permission. The tapes will be erased after they have served their purpose and all study materials will be destroyed after 15 years.

I have received a signed copy of this informed consent agreement for audio-recording.

| (The study participant’s name and date must be completed by the investigator) | |
| --- | --- |
| Study participant’s Name   \|  \|  \|  \|  \|  \|  \|  \|  \|  \|  \|  \|  \|  \|  \|  \|  \|  \|  \|  \|  \|  \| \| --- \| --- \| --- \| --- \| --- \| --- \| --- \| --- \| --- \| --- \| --- \| --- \| --- \| --- \| --- \| --- \| --- \| --- \| --- \| --- \| --- \| | |
| Study participant’s Surname   \|  \|  \|  \|  \|  \|  \|  \|  \|  \|  \|  \|  \|  \|  \|  \|  \|  \|  \|  \|  \|  \| \| --- \| --- \| --- \| --- \| --- \| --- \| --- \| --- \| --- \| --- \| --- \| --- \| --- \| --- \| --- \| --- \| --- \| --- \| --- \| --- \| --- \| | |
| Date   \| D \| D \| M \| M \| Y \| Y \| Y \| \| --- \| --- \| --- \| --- \| --- \| --- \| --- \| | Participant’s signature or thumbprint (if unable to read or write):   \|  \| \| --- \| |
| Investigator's Name   \|  \|  \|  \|  \|  \|  \|  \|  \|  \|  \|  \|  \|  \|  \|  \|  \|  \|  \|  \|  \|  \| \| --- \| --- \| --- \| --- \| --- \| --- \| --- \| --- \| --- \| --- \| --- \| --- \| --- \| --- \| --- \| --- \| --- \| --- \| --- \| --- \| --- \| | |
| Investigator's Surname   \|  \|  \|  \|  \|  \|  \|  \|  \|  \|  \|  \|  \|  \|  \|  \|  \|  \|  \|  \|  \|  \| \| --- \| --- \| --- \| --- \| --- \| --- \| --- \| --- \| --- \| --- \| --- \| --- \| --- \| --- \| --- \| --- \| --- \| --- \| --- \| --- \| --- \| | |
| Date   \| D \| D \| M \| M \| Y \| Y \| Y \| \| --- \| --- \| --- \| --- \| --- \| --- \| --- \| | Investigator's Signature   \|  \| \| --- \| |
| Witness's Name   \|  \|  \|  \|  \|  \|  \|  \|  \|  \|  \|  \|  \|  \|  \|  \|  \|  \|  \|  \|  \|  \| \| --- \| --- \| --- \| --- \| --- \| --- \| --- \| --- \| --- \| --- \| --- \| --- \| --- \| --- \| --- \| --- \| --- \| --- \| --- \| --- \| --- \| | |
| Witness's Surname   \|  \|  \|  \|  \|  \|  \|  \|  \|  \|  \|  \|  \|  \|  \|  \|  \|  \|  \|  \|  \|  \| \| --- \| --- \| --- \| --- \| --- \| --- \| --- \| --- \| --- \| --- \| --- \| --- \| --- \| --- \| --- \| --- \| --- \| --- \| --- \| --- \| --- \| | |
| Date   \| D \| D \| M \| M \| Y \| Y \| Y \| \| --- \| --- \| --- \| --- \| --- \| --- \| --- \| | Witness's Signature   \|  \| \| --- \| |

*Consent procedure should be witnessed if participant is unable to read or write.

# INFORMED CONSENT

I hereby confirm that I have been informed by the investigator, Dr ……….……about the nature, conduct, benefits and risks of clinical trial ……… I have also received, read and understood the above written information (Patient Information Leaflet, Informed Consent and audio-recording consent form) regarding the clinical trial.

I am aware that the results of the trial, including personal details regarding my sex, age, date of birth, initials and diagnosis will be anonymously processed into a trial report.

I may, at any stage, without prejudice, withdraw my consent and participation in the trial. I have had sufficient opportunity to ask questions and (of my own free will) declare myself prepared to participate in the trial.

To be completed by the patient

| **If you agree with the statements put a** ✔  **in the boxes below** | |
| --- | --- |
| 1. I confirm that I have read and understood the Patient Information Sheet for the above study, version ______ date __________ and have had the opportunity to ask questions. |  |
| 1. I confirm that any questions I have asked have been answered to my satisfaction. |  |
| 1. I understand that my participation is voluntary and that I am free to withdraw at any time, without giving any reason, and without my medical care or legal right being affected |  |
| 1. I understand that by taking part in this study my identity and location will be kept confidential. |  |
| 1. I understand that the information I share and data from my routine TB care will be accessed by the researchers. I give permission for these individuals to have access to my records. |  |
| 1. I understand that my name will not be linked with the research materials, and I will not be identified or identifiable in any reports that result from the research. |  |
| 1. **I agree to take part in the above titled study.** |  |

In addition to the above questions please put a ✔ the following box if you agree with the following statement. This statement can be opted out of and will not affect your participation.

| 1. I understand that the counselling sessions with the lay counsellor may be audio taped |  |
| --- | --- |
| 1. I understand that if I withdraw from the study, the information I have already provided will still be used for the research. |  |

| (The study participant’s name and date must be completed by the investigator) | |
| --- | --- |
| Study participant’s Name   \|  \|  \|  \|  \|  \|  \|  \|  \|  \|  \|  \|  \|  \|  \|  \|  \|  \|  \|  \|  \|  \| \| --- \| --- \| --- \| --- \| --- \| --- \| --- \| --- \| --- \| --- \| --- \| --- \| --- \| --- \| --- \| --- \| --- \| --- \| --- \| --- \| --- \| | |
| Study participant’s Surname   \|  \|  \|  \|  \|  \|  \|  \|  \|  \|  \|  \|  \|  \|  \|  \|  \|  \|  \|  \|  \|  \| \| --- \| --- \| --- \| --- \| --- \| --- \| --- \| --- \| --- \| --- \| --- \| --- \| --- \| --- \| --- \| --- \| --- \| --- \| --- \| --- \| --- \| | |
| Date   \| D \| D \| M \| M \| Y \| Y \| Y \| \| --- \| --- \| --- \| --- \| --- \| --- \| --- \| | Participant’s signature or thumbprint (if unable to read or write):   \|  \| \| --- \| |
| Investigator's Name   \|  \|  \|  \|  \|  \|  \|  \|  \|  \|  \|  \|  \|  \|  \|  \|  \|  \|  \|  \|  \|  \| \| --- \| --- \| --- \| --- \| --- \| --- \| --- \| --- \| --- \| --- \| --- \| --- \| --- \| --- \| --- \| --- \| --- \| --- \| --- \| --- \| --- \| | |
| Investigator's Surname   \|  \|  \|  \|  \|  \|  \|  \|  \|  \|  \|  \|  \|  \|  \|  \|  \|  \|  \|  \|  \|  \| \| --- \| --- \| --- \| --- \| --- \| --- \| --- \| --- \| --- \| --- \| --- \| --- \| --- \| --- \| --- \| --- \| --- \| --- \| --- \| --- \| --- \| | |
| Date   \| D \| D \| M \| M \| Y \| Y \| Y \| \| --- \| --- \| --- \| --- \| --- \| --- \| --- \| | Investigator's Signature   \|  \| \| --- \| |
| Witness's Name   \|  \|  \|  \|  \|  \|  \|  \|  \|  \|  \|  \|  \|  \|  \|  \|  \|  \|  \|  \|  \|  \| \| --- \| --- \| --- \| --- \| --- \| --- \| --- \| --- \| --- \| --- \| --- \| --- \| --- \| --- \| --- \| --- \| --- \| --- \| --- \| --- \| --- \| | |
| Witness's Surname   \|  \|  \|  \|  \|  \|  \|  \|  \|  \|  \|  \|  \|  \|  \|  \|  \|  \|  \|  \|  \|  \| \| --- \| --- \| --- \| --- \| --- \| --- \| --- \| --- \| --- \| --- \| --- \| --- \| --- \| --- \| --- \| --- \| --- \| --- \| --- \| --- \| --- \| | |
| Date   \| D \| D \| M \| M \| Y \| Y \| Y \| \| --- \| --- \| --- \| --- \| --- \| --- \| --- \| | Witness's Signature   \|  \| \| --- \| |

*Consent procedure should be witnessed if participant is unable to read or write.
