# Supplementary material for: ImPROving TB outcomes by modifying LIFE-style behaviours through a brief motivational intervention followed by short text messages (ProLife): study protocol for a randomised controlled trial
Source: Trials. 2019 Jul 26;20:457. doi: 10.1186/s13063-019-3551-9 (PMC6660690; doi:10.1186/s13063-019-3551-9)
Supplement: Supplementary file 2 — Case report form. (DOCX 58 kb) [file 13063_2019_3551_MOESM2_ESM.docx]

PROLIFE case report forms

# SCREENING AND CONSENT

| Date and time of first informed consent discussion: |  |
| --- | --- |
| STUDY INFORMATION |  |
| (Each patient must receive, read and understand this document before the start of the study) |  |
| **TRIAL TITLE:**   Improving TB outcomes by modifying life-style behaviours through a brief motivational intervention (Phase II)   **SPONSOR:** Sefako Makgatho University Principal Investigators: Prof OA Ayo-Yusuf (Sefako Makgatho University/ University of Pretoria), Prof K Siddiqi (University of York) Institution: Sefako Makgatho University  **DAYTIME AND AFTER HOURS TELEPHONE NUMBER(S):** **Daytime numbers:** 012 521 4611 **After hours:** 083 442 1970 |  |
| # INTRODUCTION  Good day.  You are invited to participate in a screening survey for a research study. Before you decide whether or not to take part, here is why we are doing the study and what it involves.  As you may know, tuberculosis (TB) is a serious concern in our country. There are many factors that influence successful TB outcomes, including HIV, treatment adherence, smoking and alcohol use. A team of local and international stakeholders and experts will develop an intervention that will target tobacco smoking, problem alcohol drinking and TB and antiretroviral treatment adherence. This study is funded by the Newton Fund and Medical Research Council. |  |
| # WHAT IS THE PROLIFE TRIAL ABOUT? WHY ARE YOU ASKED TO PARTICIPATE IN THIS STUDY?  The Prolife study is a research study on tobacco smoking, alcohol and treatment adherence in TB patients and we are inviting you to take part in this study. The study is funded by the Medical Research Council-Newton Foundation and is approved by the ethics committees of Sefako Makgatho University, the University of York, the University of Pretoria, the University of Witwatersrand and South African Medical Research Council in South Africa. Here, we explain why we are doing the Prolife Study and what it will involve. This will help you to decide whether to participate in this study. You are free to choose whether or not to participate in this study.  The purpose of this screening procedure is to collect information about you to determine whether or not you qualify to take part in the study. This screening will involve you answering some questions on your current health, smoking and alcohol drinking behaviour. A research assistant will also examine your medical records during this screening procedure. In order to qualify, you must be an adult with pulmonary tuberculosis who smokes tobacco or who drinks alcohol. |  |
| # WHAT WILL I HAVE TO DO?  You will have to give consent for the screening including the examining of your medical records. If you agree to the screening procedure (or to participate) you will be asked a series of questions to determine your eligibility. These questions include information about your current health, treatment and smoking and drinking behaviour. This screening procedure will take approximately 10 minutes. |  |
| # ARE THERE ANY RISKS OR DISCOMFORT INVOLVED?  There are no direct risks to your health but you might feel uncomfortable in answering certain questions. If you do, you may withdraw from the screening at any time. |  |
| # IF I TAKE PART WILL MY INFORMATION BE KEPT CONFIDENTIAL?  All the information we collect from you on the paper documents will be kept confidential on secure computers and in locked filing cabinets in locked offices and transferred safely to the country’s coordinating centre at Sefako Makgatho University to be secured.  Data that will be collected using the mobile phone, including your phone number, will be securely stored on the mobile phone and an online storage space. Only authorized researchers and personnel will be able to access the information. After we are finished collecting data, your data will be de-identified (all information that might be traced to you will be removed) and then it will be shared with the researchers working on this project.  The research staff will ensure that your identity and location in this study will remain strictly confidential. However, there is a slight risk that your information and number might get stolen by malicious people and used inappropriately.  Make sure that you provide your own phone numbers or the number of a person who you feel comfortable with to share information about TB, alcohol, and tobacco.  The results of the study, including data, may be published for scientific purposes but will not give your name or include any identifiable references to you.  The study materials will be kept for 15 years; after which time they will be destroyed. |  |
| # WHAT ARE THE POSSIBLE BENEFITS OF TAKING PART IN THE STUDY?  You may qualify to participate in the research study and it is hoped that the information collected from the study will help the researches to develop an intervention which may benefit your health. |  |
| # REIMBURSEMENT  You will not receive reimbursement for the screening. You will also not be charged for the screening. |  |
| # HAS THE STUDY RECEIVED ETHICAL APPROVAL?  This Protocol was submitted to the Research Ethics Committees of the relevant Universities: Sefako Makgatho University; University of York; University of Witwatersrand; the University of Pretoria, the Medical Research Council; and the University of the Free State. The study has been structured in accordance with the Declaration of Helsinki (last update: October 2013), which deals with the recommendations guiding doctors in biomedical research involving human/subjects. A copy of the Declaration may be obtained from the investigator should you wish to review it. If you have any queries related to ethics, you may contact: |  |
| # WHERE CAN I OBTAIN ADDITIONAL INFORMATION? If I have any questions concerning this study, I should contact: · M Malefo, cell: 072 6412747 · Prof Olu Omole, tel: 016 950 6192 cell: 076 472 1289 (Sedibeng district) · Prof John Tumbo, tel: 012 521 4314 or cell: 082 885 8332 (Bojanala district) If I have any queries related to ethics, I should contact: 1. Dr C.Baker at Sefako Makgatho Health Sciences University (telephone number: 012 521 5617); 2. Dr S Holland at the University of York (telephone number: 019 0432 4031); 3. University of the Witwatersrand Prof P Cleaton Jones, Chair, Tel 011 717 2301, Email: peter.cleaton-jones1@wits.ac.za Ms Z Ndlovu, Administrative Officer, Email: zanele.ndlovu@wits.ac.za Mr Rhulani Mkansi, Administrative Officer, Email: rhulani.mkansi@wits.ac.za Mr Lebo Moeng, Administrative Officer, Email: lebo.moeng@wits.ac.za Tel: 011 717 2700/2656/1234/1252 4. Professor W Van Staden at the University of Pretoria (telephone numbers: 012 354 1677/ 012 354 1330); 5. Professor D du Toit at the South African Medical Research Council (telephone number: 021 938 0687). |  |
| CONSENT INFORMATION |  |
| Is the participant able to read and write? | No [0] Yes [1] |
| CONSENT TO PARTICIPATE IN THIS STUDY (To be completed by those who are literate) |  |
| I have read or had read to me in a language that I understand the above information before signing this consent form. The content and meaning of this information have been explained to me. I have been given opportunity to ask questions and am satisfied that they have been answered satisfactorily. I understand that if I do not participate it will not alter my management in any way. I hereby volunteer to take part in this screening stage of the study. |  |
| I have received a signed copy of this informed consent agreement. | No [0] Yes [1] |
| If you do not receive a signed copy of the informed consent agreement, end the interview. |  |
| CONSENT TO EXAMINE PATIENT’S MEDICAL RECORD (To be completed by those who are literate) |  |
| I have read or had read to me in a language that I understand the information about this study before signing this consent form. The content and meaning of this information have been explained to me. I have been given opportunity to ask questions and I am satisfied that they have been answered satisfactorily. I understand that if I do not give consent for my medical records to be examined by the researchers, it will not alter my management in any way. I hereby consent to my medical records being examined at this stage of the study. |  |
| I have received a signed copy of this informed consent agreement. | No [0] Yes [1] |
| If you did not receive a signed copy of the informed consent agreement, end the interview. |  |
| VERBAL PATIENT INFORMED CONSENT (To be completed by those who are illiterate) |  |
| (This section is only applicable to participants who are unable to read and write)  I, the undersigned, _________________________, have read and have explained fully to the study participant’s, named _________________and/or his/her relative, the patient information leaflet, which has indicated the nature and purpose of the study in which I have asked the patient to participate. The explanation I have given has mentioned both the possible risks and benefits of the study and the alternative treatments available for his/her illness. The patient indicated that he/she understands that he/she will be free to withdraw from the study at any time for any reason and without jeopardizing his/her treatment.  The patient consents to the obtaining of information from his/her TB file to determine the type of TB she/he has and to determine for how long she/he has been on treatment. |  |
| I hereby certify that the patient has agreed to participate in this study. | No [0] Yes [1] |
| If the participant did not agree to participate in the study, end the interview. |  |
| VERBAL CONSENT TO EXAMINE MEDICAL RECORDS (To be completed by those who are illiterate) |  |
| (This section is only applicable to participants who are unable to read and write) I, the undersigned, _________________________, have read and have explained fully to the study participant’s, named _________________and/or his/her relative, the patient information leaflet, which has indicated the nature and purpose of the study in which I have asked the patient to consent that his/her medical record be examined. The patient indicated that he/she understands that he/she will be free to withdraw her/his consent at any time, for any reason and without jeopardizing his/her treatment. The patient consents to the obtaining of information from his/her TB file to determine the type of TB she/he has and to determine for how long she/he has been on treatment. |  |
| I have received a signed copy of this informed consent agreement. | No [0] Yes [1] |
| If you did not receive a signed copy of the verbal consent agreement, end the interview. |  |
| SCREENING TOOL |  |
| PERSONAL AND TB INFORMATION |  |
| What was your age in years on your last birthday? |  |
| The participant is too young to participate in the study. Please end the interview. |  |
| Sex | Male [0] Female [1] |
| Does the patient have pulmonary TB? | No [0] Yes [1] |
| If “No” please end the interview. |  |
| Has the patient been on treatment for more than four weeks? | No [0] Yes [1] |
| If “Yes” please end the interview. |  |
| Do you speak and understand any of the following languages: Zulu or Setswana or Sesotho or English? | No [0] Yes [1] |
| If not, please end the interview. |  |
| CONTACT DETAILS |  |
| Do you have easy access to a functional cell phone? This will allow us to SMS you supportive messages and send you reminders regarding appointments. | No [0] Yes [1] |
| If No easily accessible cell phone, please end the interview |  |
| Collect Contact Number for Study Participant |  |
| Enter phone number |  |
| Enter phone number again |  |
| Alternative cell phone number |  |
| Enter alternative cell phone number |  |
| Enter alternative cell phone number again. |  |
| SMOKING AND DRINKING HISTORY |  |
| In the past month, have you smoked tobacco on a daily basis, less than daily basis or not at all? | Daily [1] Less than daily [2] Not at all [0] |
| Have you had a drink containing alcohol in the past 12 months? alcoholic drink in past 12 months | No [0] Yes [1] |
| AUDIT |  |
| Below is a list of questions about your drinking behaviour. Please circle the score (0-4) that best reflects your behaviour.  In the past 12 months: |  |
| How often do you have a drink containing alcohol? | Never [0] Monthly or less [1] 2 to 4 times a month [2] 2 to 3 times a week [3] 4 or more times a week [4] |
| How many drinks containing alcohol do you have on a typical day when you are drinking? (Please note that one drink is equivalent to one can or bottle of beer, cider or cooler, one glass of wine, or one tot of spirits). | 1 or 2 [0] 3 or 4 [1] 5 or 6 [2] 7 to 9 [3] 10 or more [4] |
| How often do you have six or more drinks on one occasion? | Never [0] Less than monthly [1] Monthly [2] Weekly [3] Daily or almost daily [4] |
| How often during the last year have you found that you were not able to stop drinking once you have started? | Never [0] Less than monthly [1] Monthly [2] Weekly [3] Daily or almost daily [4] |
| How often during the last year have you failed to do what was normally expected from you because of drinking? | Never [0] Less than monthly [1] Monthly [2] Weekly [3] Daily or almost daily [4] |
| How often during the last year have you needed a first drink in the morning to get yourself going after a heavy drinking session? | Never [0] Less than monthly [1] Monthly [2] Weekly [3] Daily or almost daily [4] |
| How often during the last year have you had a feeling of guilt or remorse after drinking? | Never [0] Less than monthly [1] Monthly [2] Weekly [3] Daily or almost daily [4] |
| How often during the last year have you been unable to remember what happened the night before because you had been drinking? | Never [0] Less than monthly [1] Monthly [2] Weekly [3] Daily or almost daily [4] |
| Have you or someone else been injured as a result of your drinking? | No [0] Yes, but not in the last year [2] Yes, during the last year [4] |
| Has a relative, friend, or a doctor or another health worker been concerned about your drinking or suggested you cut down? | No [0] Yes, but not in the last year [2] Yes, during the last year [4] |
| RCT CONSENT (Only to be completed by those who are eligible) |  |
| DATE AND TIME OF FIRST INFORMED CONSENT DISCUSSION: |  |
| Consent procedure should be witnessed if participant is unable to read or write. |  |
| STUDY INFORMATION |  |
| (Each patient must receive, read and understand this document before the start of the study) |  |
| **TRIAL TITLE:** Improving TB outcomes by modifying life-style behaviours through a brief motivational intervention (Phase II) **SPONSOR:** Sefako Makgatho University **Principal Investigators:** Prof OA Ayo-Yusuf (Sefako Makgatho University/ University of Pretoria), Prof K Siddiqi (University of York) **Institution:** Sefako Makgatho University  **DAYTIME AND AFTER HOURS TELEPHONE NUMBER(S):** **Daytime numbers:** 012 521 4611 **After hours:** 083 442 1970 |  |
| # INTRODUCTION You have been found to be eligible and, as such, are invited to volunteer for this research study. This information leaflet is to help you to decide if you would like to participate. Before you agree to take part in this study you should fully understand what is involved. If you have any questions, which are not fully explained in this leaflet, do not hesitate to ask the investigator. You should not agree to take part unless you are completely happy about all the procedures involved. In the best interests of your health, it is strongly recommended that you discuss with or inform your personal doctor of your possible participation in this study, wherever possible. |  |
| # WHAT IS THE PURPOSE OF THE RESEARCH TRIAL? As you may know, tuberculosis (TB) is a serious concern in our country. There are many factors that influence successful TB outcomes, including HIV, treatment adherence, smoking and alcohol use. A team of local and international stakeholders and experts developed an intervention that will target tobacco smoking, problem alcohol drinking and TB and antiretroviral treatment adherence. |  |
| # WHAT IS THE PROLIFE TRIAL ABOUT? WHY ARE YOU ASKED TO PARTICIPATE IN THIS STUDY? The Prolife study is a research study on tobacco smoking, alcohol and treatment adherence in TB patients. The study is funded by the Medical Research Council-Newton Foundation and was approved by the ethics committees of Sefako Makgatho University, the University of York, the University of Pretoria, the University of Witwatersrand and South African Medical Research Council in South Africa. Here, we explain why we are doing the Prolife Study and what it will involve. This will help you to decide whether to participate in this study. You are free to choose whether or not to participate in this study.  All participants in this trial will receive the usual treatment for TB and will be seen by the nurse as normal. Around half of the participants will receive some additional sessions (the Prolife package) as well as this. This is decided at random and you will not be able to request to receive this. It is important to realise that no participant will receive anything less than the usual TB treatment as advised by all recognised guidelines.  Those participants who are given the Prolife package will receive three extra sessions one month apart with a lay health worker. These sessions will focus on looking at ways of motivating you to reduce your smoking, reduce your alcohol and to take your medications properly. You will also receive text messages to your phone to reinforce these sessions. Participants who are given the Prolife package will therefore be asked to provide their phone numbers. The sessions with the lay counselors may be recorded. This will be done strictly to study whether the counselor how well the counselor manages the counseling sessions. Your personal information discussed will be kept strictly confidential. All participants will have 3 sessions with a researcher who will ask you questions about your background, your general health, your smoking and alcohol habits and how well you take your medications. Each session is expected not to last more than 25 minutes. By consenting to take part in this study you are also consenting to allow this researcher access to your TB treatment record. This information will be kept anonymous and confidential by the research team. |  |
| # WHAT IS THE DURATION OF THIS TRIAL? If you decide to take part you will be one of approximately 720 patients. The study will last for up to 6 months. You will be asked to visit the investigator 3 times as per the following schedule: at the beginning, at 3 months and at 6 months. |  |
| # I UNDERSTAND THAT IF I DO NOT WANT TO PARTICIPATE IN THIS STUDY, I WILL STILL RECEIVE STANDARD TREATMENT FOR MY ILLNESS. |  |
| # I MAY AT ANY TIME WITHDRAW FROM THIS STUDY. |  |
| # HAS THE TRIAL RECEIVED ETHICAL APPROVAL? This Protocol was submitted to the Research Ethics Committees of the relevant Universities: Sefako Makgatho University; University of York; University of Witwatersrand; the University of Pretoria, the Medical Research Council; and the University of the Free State. The study has been structured in accordance with the Declaration of Helsinki (last update: October 2013), which deals with the recommendations guiding doctors in biomedical research involving human/subjects. A copy of the Declaration may be obtained from the investigator should you wish to review it. If you have any queries related to ethics, you may contact: |  |
| # WHERE CAN I OBTAIN ADDITIONAL INFORMATION? If I have any questions concerning this study, I should contact Mrs M Malefo, tel: 012 521 4611 or cell: 0726472741 Prof Olu Omole, tel: 016 950 6192 cell: 076 472 1289 (Sedibeng district Prof John Tumbo, tel: 012 521 4314 or cell: 082 885 8332 (Bojanala district If I have any queries related to ethics, I should contact: 1. Dr C.Baker at Sefako Makgatho Health Sciences University (telephone number: 012 521 5617); 2. Dr S Holland at the University of York (telephone number: 019 0432 4031); 3. University of the Witwatersrand Prof P Cleaton Jones, Chair, Tel 011 717 2301, Email: peter.cleaton-jones1@wits.ac.za Ms Z Ndlovu, Administrative Officer, Email: zanele.ndlovu@wits.ac.za Mr Rhulani Mkansi, Administrative Officer, Email: rhulani.mkansi@wits.ac.za Mr Lebo Moeng, Administrative Officer, Email: lebo.moeng@wits.ac.za Tel: 011 717 2700/2656/1234/1252 4. Professor W Van Staden at the University of Pretoria (telephone numbers: 012 354 1677/ 012 354 1330); 5. Professor D du Toit at the South African Medical Research Council (telephone number: 021 938 0687). |  |
| # IF I TAKE PART WILL MY INFORMATION BE KEPT CONFIDENTIAL? All the information we collect from you on the paper documents and during audio recordings will be kept confidential on secure computers and in locked filing cabinets in locked offices and transferred safely to the country’s coordinating centre at Sefako Makgatho University to be secured.  Data that will be collected using the mobile phone, including your phone number, will be securely stored on the mobile phone and an online storage space. Only authorized researchers and personnel will be able to access the information. After we are finished collecting data, your data will be de-identified (all information that might be traced to you will be removed) and then it will be shared with the researchers working on this project.  Your phone number will also be stored on a short text message (SMS) company’s secured sever. This company will assist us to send you appointment reminders and messages about TB, alcohol, and tobacco. The companies and the research team are not allowed to use your number for any other purposes.  The research staff will ensure that your identity and location in this study will remain strictly confidential. However, there is a slight risk that your information and number might get stolen by malicious people and used inappropriately.  Make sure that you provide your own phone numbers or the number of a person who you feel comfortable with to share information about TB, alcohol, and tobacco.  The results of the study, including data, may be published for scientific purposes but will not give your name or include any identifiable references to you.  The study materials will be kept for 15 years; after which time they will be destroyed. |  |
| # WHAT ARE YOUR RIGHTS AS A PARTICIPANT IN THIS TRIAL? Your participation in this trial is entirely voluntary and you can refuse to participate or stop at any time without stating any reason. Your withdrawal will not affect your access to other medical care. The investigator retains the right to withdraw you from the study if it is considered to be in your best interest. If it is detected that you did not give an accurate history or did nor follow the guidelines of the trial and the regulations of the trial facility, you may be withdrawn from the trial at any time. |  |
| # IS ALTERNATIVE TREATMENT AVAILABLE? Alternative treatment in the form of seeing the nurse and having the usual TB treatment given to all patients is available and will help to treat your TB. If you decide not to take part in this study you will still receive all of this usual treatment. |  |
| # WHAT ARE THE RISKS INVOLVED IN THIS TRIAL? MAY ANY OF THESE TRIAL PROCEDURES RESULT IN DISCOMFORT OR INCONVENIENCE. There are no technical procedures involved. However, the follow-up sessions will take some of your time (about 45 minutes of your time: 25 minutes for questions about your background, your general health, your smoking and alcohol habits and how well you take your medications. Another 20 minutes for counselling, if you are allocated to receive counselling from the Lay health worker. This does not include time involved to travel to and from the venue). |  |
| # WHAT ARE THE BENEFITS TO YOU? We hope that we are able to help you in three main ways (one or all of these may apply to you): we hope we can help you to reduce the amount of tobacco you are smoking, the amount of alcohol you consume and, if appropriate, we hope that we can help you to ensure that you improve how you take your TB and/or HIV medication. We think that doing one or all of these might help to improve your recovery from TB and if this is found to be the case, the Prolife package may be used to help many patients with TB in the future. |  |
| # REIMBURSEMENT  To cover your travel expenses for the extra visits, you will be reimbursed to the value of 60 Rand per follow-up session. |  |
| # INFORMED CONSENT  I hereby confirm that I have been informed by the investigator, Dr ……….……about the nature, conduct, benefits and risks of clinical trial ……… I have also received, read and understood the above written information (Patient Information Leaflet, Informed Consent and audio-recording consent form) regarding the clinical trial.  I am aware that the results of the trial, including personal details regarding my sex, age, date of birth, initials and diagnosis will be anonymously processed into a trial report.  I may, at any stage, without prejudice, withdraw my consent and participation in the trial. I have had sufficient opportunity to ask questions and (of my own free will) declare myself prepared to participate in the trial.  To be completed by the patient |  |
| AUDIO-RECORDING CONSENT |  |
| CONSENT FOR AUDIO-RECORDING OF COUNSELLING SESSION WITH LAY HEALTH WORKER |  |
| I have read or had read to me in a language that I understand, the above information regarding this trial and the need for an audio recording of the interview before signing this audio-recording consent form. The content and meaning of this information have been explained to me. I have been given opportunity to ask questions and I am satisfied that they have been answered satisfactorily.  I understand that I do not have to give consent to this audio recording. I further understand that I can request that the tape recorder be turned off at any time and may request that the tape or any portion thereof be erased.  I may terminate this permission to audio-tape at any time. The contents of these taped sessions are confidential and the information will not be shared outside the context of this research. The tapes will be stored in a secure location and will not be used for any other purpose without my explicit written permission. The tapes will be erased after they have served their purpose and all study materials will be destroyed after 15 years. |  |
| I have received a signed copy of this informed consent agreement for audio-recording. | No [0] Yes [1] |
| Do not audio record MI sessions if participant did not consent to it. |  |
| CONSENT TO STUDY PARTICIPATION |  |
| If you agree with the statements put a ✔ in the boxes below |  |
| I confirm that I have read and understood the Patient Information Sheet for the above study, version ______ date __________ and have had the opportunity to ask questions. | No [0] Yes [1] |
| If the participant did not understand the Patient Information Sheet, clarify any question they may have or end interview. |  |
| I confirm that any questions I have asked have been answered to my satisfaction. | No [0] Yes [1] |
| Clarify any questions that the participant might have. If they still select No, end interview. |  |
| I understand that my participation is voluntary and that I am free to withdraw at any time, without giving any reason, and without my medical care or legal right being affected | No [0] Yes [1] |
| If the patient selected no, clarify their voluntary participation or end interview. |  |
| I understand that by taking part in this study my identity and location will be kept confidential. | No [0] Yes [1] |
| If the participant selected No, clarify their confidentiality or end interview. |  |
| I understand that the information I share and data from my routine TB care will be accessed by the researchers. I give permission for these individuals to have access to my records. | No [0] Yes [1] |
| If the participant selected No, clarify the access to their TB information or end interview. |  |
| I understand that my name will not be linked with the research materials, and I will not be identified or identifiable in any reports that result from the research. | No [0] Yes [1] |
| If the participant selected No, try to explain or end interview. |  |
| I agree to take part in the above titled study. | No [0] Yes [1] |
| If the participant selected No, end interview. |  |
| In addition to the above questions please put a ✔ the following box if you agree with the following statement. This statement can be opted out of and will not affect your participation. |  |
| I understand that the counselling sessions with the lay counsellor may be audio taped | No [0] Yes [1] |
| If the participant selected No, then you should not allow the MI sessions with the counsellor to be recorded. |  |
| I understand that if I withdraw from the study, the information I have already provided will still be used for the research. | No [0] Yes [1] |
| If the participant selected No, clarify or end interview. |  |
| I have received a signed copy of this informed consent agreement. | No [0] Yes [1] |
| If you do not get a signed copy of the informed consent agreement, end interview. |  |

# BASELINE PARTICIPANT QUESTIONS

| DEMOGRAPHIC AND SOCIOECONOMIC INFORMATION |  |
| --- | --- |
| I'm now going to ask you about your socioeconomic and demographic status because your status might influence your TB treatment success. This section will include questions about your marital status, education, employment, and household luxuries. The answers to these questions will be kept completely confidential and will not be used in any way outside of the study or to harm you. You are free to decline answering to any of these questions. |  |
| What is your current marital status? | Married or living together [1] Divorced/separated [2] Widowed [3] Never married and never lived together. [4] Declined to answer [5] |
| EDUCATION AND EMPLOYMENT |  |
| What is the highest level of education you have completed? | No education [0] Grades 1-5 [1] Grades 6-7 [2] Grades 8-11 [3] Grade 12 [4] Higher [5] Declined to answer [6] |
| What is your employment status? Which one of these best describes you? | Self-employed (full-time) [1] Employed full-time (30 hrs a week or more) [2] Employed part-time (less than 30 hrs a week) [3] Retired [4] Unemployed (but able to work) [5] Unable to work because of long-term disability or ill health [6] Full-time student [7] Caring from my home and family/doing household work/housewife [8] Occasional work (“piece job”) [9] Declined to answer [10] |
| # INSTRUCTION TO THE FIELDWORKER  The following is an unprompted question, i.e. the fieldworker does not say the name of each type of mine.  Do not read the list of mines to the participant. Instead, ask the participant to tell you in which mine(s) they worked and select the "Yes" answer for the mines that were mentioned.  If the participant did not mention a mine on the list of options then you have to select "Yes" to the option "Other" and describe the mine.  If the participant indicated that they worked or spent time in a mine, then you have to select at least one of the options. |  |
| Have you ever worked or spent time in mines? | No [0] Yes [1] Declined to answer [3] |
| Types of mines |  |
| Which type of mine have you worked in? |  |
| Coal | No [0] Yes [1] |
| Diamond | No [0] Yes [1] |
| Gold | No [0] Yes [1] |
| Platinum and palladium | No [0] Yes [1] |
| Chromium | No [0] Yes [1] |
| Uranium | No [0] Yes [1] |
| Manganese | No [0] Yes [1] |
| Other | No [0] Yes [1] |
| Please specify. |  |
| SOCIOECONOMIC STATUS |  |
| Does your household have any of the following items in working condition? |  |
| Household Items |  |
| A radio | No [0] Yes [1] Declined to answer [2] |
| A television | No [0] Yes [1] Declined to answer [3] |
| A landline telephone | No [0] Yes [1] Declined to answer [2] |
| A desktop or laptop computer | No [0] Yes [1] Declined to answer [2] |
| A refrigerator | No [0] Yes [1] Declined to answer [2] |
| A vacuum cleaner or floor polisher | No [0] Yes [1] Declined to answer [2] |
| A microwave oven | No [0] Yes [1] Declined to answer [2] |
| An electric or gas stove | No [0] Yes [1] Declined to answer [2] |
| A washing machine | No [0] Yes [1] Declined to answer [2] |
| Let us speak about your household and what it can afford. In the past month, how many days did you or people in the household go to bed hungry because there was no food to eat? | 0 days [1] 1-7 days [2] More than 7 days [3] Declined to answer [4] |
| SOCIAL HISTORY AND LIFESTYLE FACTORS |  |
| SMOKING HISTORY (Only asked to those who smoke) |  |
| On the days that you smoke, how soon after you wake up do you have your first cigarette? | Within 5 minutes [3] 6- 30 minutes [2] 31-60 minutes [1] After 60 minutes [0] |
| SMOKING DURATION |  |
| How old (years) were you when you first started using tobacco? |  |
| For how long have you SMOKED tobacco? |  |
| Number of years: |  |
| Number of months: |  |
| TOBACCO FORM |  |
| In which form do you use tobacco? |  |
| Manufactured cigarettes | No [0] Yes [1] |
| Manufactured cigarettes |  |
| If yes, in the past 7 days, on how many days did you smoke manufactured cigarettes? |  |
| If yes, what is the average number of manufactured cigarettes that you smoke EACH DAY? |  |
| Hand-rolled cigarettes | No [0] Yes [1] |
| Hand-rolled cigarettes |  |
| If yes, in the past 7 days, on how many days did you smoke hand-rolled cigarettes? |  |
| If yes, what is the average number of hand-rolled cigarettes that you smoke EACH DAY? |  |
| Pipe | No [0] Yes [1] |
| Pipe |  |
| If yes, in the past 7 days, on how many days did you smoke pipe? |  |
| If yes, what is the average number of times that you smoke pipe EACH DAY? |  |
| If yes, how long does one session (without break) last? |  |
| Hours |  |
| and minutes |  |
| Cigars, cheroots or cigarillos | No [0] Yes [1] |
| Cigars |  |
| If yes, in the past 7 days, on how many days did you smoke cigars, cheroots or cigarillos? |  |
| If yes, what is the average number of times you used this form of tobacco EACH DAY? |  |
| Water pipe | No [0] Yes [1] |
| Water pipe |  |
| If yes, in the past 7 days, on how many days did you use water pipe/hubbly bubbly? |  |
| If yes, what is the average number of water pipe sessions EACH DAY? |  |
| If yes, how long does one session (without break) last? |  |
| Hours |  |
| and minutes |  |
| Other tobacco | No [0] Yes [1] |
| If yes, please specify |  |
| If yes, in the past 7 days, on how many days did you smoke cigarettes? |  |
| If yes, what is the average number of cigarettes that you smoke EACH DAY? |  |
| It seems like the participant did not understand the question asking them in which form they use tobacco.   The participant indicated during screening that they are smoking. They should therefore have mentioned at least one of the items on the list or you should have selected Other Tobacco and entered the item that the participant mentioned.   Please clarify the question and ask them again. |  |
| Which statement best describes the rules about tobacco smoking INSIDE YOUR HOME? | No one is allowed to smoke anywhere, ever [1] Smoking is allowed in some places or at sometimes [2] Smoking is permitted anywhere, there are no rules [3] |
| ATTEMPTED TO QUIT |  |
| Have you ever attempted to quit smoking in the past? | No [0] Yes [1] |
| If yes, how many times? |  |
| LAST ATTEMPT |  |
| If yes, when did you attempt to quit last time? |  |
| Years ago |  |
| and number of months ago: |  |
| DURATION ABSTAINED |  |
| What was the longest duration that you stayed abstinent in previous quit attempts? |  |
| Years: |  |
| and number of months: |  |
| and the number of days: |  |
| How likely is it that you will TRY TO QUIT smoking completely and permanently in the next three months? | I definitely will not [0] I probably will not [1] I probably will [2] I definitely will [3] |
| How likely is it that you WILL QUIT smoking completely and permanently in the next three months? | I definitely will not [0] I probably will not [1] I probably will [2] I definitely will [3] |
| INSTRUCTION TO THE FIELDWORKER  The following is an unprompted question.  Do not read the list of methods to the participant. Instead, ask the participant to tell you about the methods they used and select the "Yes" answer for the methods that were mentioned.  If the participant did not mention a method on the list of options then you have to select "Yes" to the option "Other" and describe the method provided by the participant.  If the participant indicated that they did use a method in the past to help them stop smoking, then you have to select at least one of the options.  For example, the patient answers that she/he went to the chemist and bought nicotine gum. Then the fieldworker needs to select "Yes" for "Nicotine gum or another nicotine replacement product” from the list of options. |  |
| METHODS TO STOP SMOKING |  |
| Have you ever used any methods in the past to help you stop smoking tobacco in the past 3 months? | No [0] Yes [1] |
| If yes, please describe |  |
| Counselling by a nurse or doctor of counsellor | No [0] Yes [1] |
| Nicotine gum or another nicotine replacement product | No [0] Yes [1] |
| Traditional healer | No [0] Yes [1] |
| Quit helpline | No [0] Yes [1] |
| Other prescribed tobacco cessation treatment (other than Nicotine Replacement therapy) | No [0] Yes [1] |
| Other | No [0] Yes [1] |
| If other, please describe |  |
| How much did you spend (in Rands), out of your pocket, on any of the methods to help you stop smoking in the past 3 months? R |  |
| How much have you spent on average per week on cigarettes over the past 3 months? R |  |
| SMOKELESS TOBACCO USE |  |
| In the past month, have you used smokeless tobacco (Snuff) on a daily basis, less than daily basis or not at all? | Not at all [0] Daily [1] Less than Daily [2] |
| SMOKELESS TOBACCO USE |  |
| DURATION |  |
| How old (years) were you when you first started using smokeless tobacco? |  |
| For how long have you used smokeless tobacco? [Complete number of years AND number of months (i.e. not OR)] |  |
| Years: |  |
| Months: |  |
| FORM |  |
| In which form do you use smokeless tobacco? (tick any that apply), [coded as 0 or 1 for each question] |  |
| Snuff (by mouth) | No [0] Yes [1] |
| Snuff (by nose) | No [0] Yes [1] |
| Chewing tobacco leaves | No [0] Yes [1] |
| Other | No [0] Yes [1] |
| If other, please specify: |  |
| TOBACCO SMOKE EXPOSURE |  |
| In the past 30 days, about how many days would you say you were in a place where someone smoked close to you (no complete physical barrier, i.e. smoke got to you)? |  |
| At home | 7 days or less [0] More than 7 days [1] |
| At work | 7 days or less [0] More than 7 days [1] |
| Cafes/restaurants | 7 days or less [0] More than 7 days [1] |
| Shebeens, bars or clubs | 7 days or less [0] More than 7 days [1] |
| Bus/train/taxi/ vehicle | 7 days or less [0] More than 7 days [1] |
| Shops/shopping mall | 7 days or less [0] More than 7 days [1] |
| HELP TO STOP DRINKING (Only asked to those who drink) |  |
| # HELP TO STOP SMOKING |  |
| Have you ever used any methods in the past to help you stop drinking alcohol in the past 3 months? | No [0] Yes [1] |
| If yes, please describe |  |
| How much have you spent (in Rands) on average per week on alcohol over the past 3 months? |  |
| Now we would like to ask you some questions about how your mood has been lately. For each of the following statements, please check the box that best describes how often you felt or behaved this way during the past week. (CESD 10) |  |
| CESD 10 |  |
| I was bothered by things that usually don’t bother me. | Rarely or None of the Time (Less than 1 day) [1] Some or a Little of the Time (1-2 days) [2] Occasionally or a Moderate Amount of the Time (3-4 days) [3] Most or All of the Time (5-7 days) [4] |
| I had trouble keeping my mind on what I was doing. | Rarely or None of the Time (Less than 1 day) [1] Some or a Little of the Time (1-2 days) [2] Occasionally or a Moderate Amount of the Time (3-4 days) [3] Most or All of the Time (5-7 days) [4] |
| I felt depressed. | Rarely or None of the Time (Less than 1 day) [1] Some or a Little of the Time (1-2 days) [2] Occasionally or a Moderate Amount of the Time (3-4 days) [3] Most or All of the Time (5-7 days) [4] |
| I felt that everything I did was an effort. | Rarely or None of the Time (Less than 1 day) [1] Some or a Little of the Time (1-2 days) [2] Occasionally or a Moderate Amount of the Time (3-4 days) [3] Most or All of the Time (5-7 days) [4] |
| I felt hopeful about the future. | Rarely or None of the Time (Less than 1 day) [4] Some or a Little of the Time (1-2 days) [3] Occasionally or a Moderate Amount of the Time (3-4 days) [2] Most or All of the Time (5-7 days) [1] |
| I felt fearful. | Rarely or None of the Time (Less than 1 day) [1] Some or a Little of the Time (1-2 days) [2] Occasionally or a Moderate Amount of the Time (3-4 days) [3] Most or All of the Time (5-7 days) [4] |
| My sleep was restless. | Rarely or None of the Time (Less than 1 day) [1] Some or a Little of the Time (1-2 days) [2] Occasionally or a Moderate Amount of the Time (3-4 days) [3] Most or All of the Time (5-7 days) [4] |
| I was happy. | Rarely or None of the Time (Less than 1 day) [4] Some or a Little of the Time (1-2 days) [3] Occasionally or a Moderate Amount of the Time (3-4 days) [2] Most or All of the Time (5-7 days) [1] |
| I felt lonely. | Rarely or None of the Time (Less than 1 day) [1] Some or a Little of the Time (1-2 days) [2] Occasionally or a Moderate Amount of the Time (3-4 days) [3] Most or All of the Time (5-7 days) [4] |
| I could not get “going”. | Rarely or None of the Time (Less than 1 day) [1] Some or a Little of the Time (1-2 days) [2] Occasionally or a Moderate Amount of the Time (3-4 days) [3] Most or All of the Time (5-7 days) [4] |
| HEALTH ECONOMICS |  |
| You should exclude current care that you are receiving in your answers to these questions |  |
| Have you visited any health facility for TB symptoms and/or initial treatment in the past 3 months? | No. [0] Yes [1] |
| FACILITY |  |
| Did you visit a public clinic or health centre? | No [0] Yes [1] |
| Public clinic or facility |  |
| How many times did you visit this facility? |  |
| Indicate which health worker you were seen by at the clinic for the reason of your visit: |  |
| Nurse | No [0] Yes [1] |
| Doctor | No [0] Yes [1] |
| How much did you pay (in Rands) in total including travel per visit? R |  |
| How many hours did it take per visit to travel from home and back? |  |
| Of this time, how many hours did the total consultation for the reason of your visit take? |  |
| Of the total consultation time, how much time (in minutes) did you spend with the doctor? |  |
| Did you visit a public hospital? | No [0] Yes [1] |
| Public hospital |  |
| How many times did you visit this facility? |  |
| Indicate which health worker you were seen by at the hospital for the reason of your visit: |  |
| Nurse | No [0] Yes [1] |
| Doctor | No [0] Yes [1] |
| How much did you pay (in Rands) in total including travel per visit? R |  |
| How many hours did it take per visit to travel from home and back? |  |
| Of this time, how many hours did the total consultation for the reason of your visit take? |  |
| Of the total consultation time, how much time (in minutes) did you spend with the doctor? |  |
| Did you have to stay overnight at any of these visits? | No [0] Yes [1] |
| If Yes, how many nights did you spend per visit? |  |
| EQ-5D-3L |  |
| # INTRODUCTION TO EQ-5D  We are trying to find out what you think about your health. I will first ask you a few brief and simple questions about your own state of health today. I will then ask you to do a rather different task that involves rating your health on a measuring scale. I will explain the tasks fully as I go along but please interrupt me if you do not understand something or if things are not clear to you. Please also remember that there are no right or wrong answers. We are interested here only in your personal view. |  |
| First I am going to read out some questions. Each question has a choice of three answers. Please tell me which answer best describes your own state of health today.  Do not choose more than one answer in each group of questions.  [Note for administrator: it may be necessary to remind the respondent regularly that the timeframe is today] |  |
| MOBILITY |  |
| First I'd like to ask you about mobility. |  |
| Question 1: Would you say you have … | No problems in walking about? [0] Some problems in walking about? [1] Are you confined to bed? [2] |
| So, would you say you have no problems in walking about, some problems in walking about or are you confined to bed?  (Note for administrator: tick the appropriate box on the EQ-5D questionnaire) |  |
| SELF-CARE |  |
| Next I'd like to ask you about self-care. |  |
| Question 2: Would you say you have … | No problems with self-care? [0] Some problems washing or dressing yourself? [1] Are you unable to wash or dress yourself? [2] |
| So, would you say you have no problems with self-care, some problems washing or dressing yourself or are you unable to wash or dress yourself?  (Note for administrator: tick the appropriate box on the EQ-5D questionnaire) |  |
| USUAL ACTIVITIES |  |
| Next I'd like to ask you about usual activities, for example work, study, housework, family or leisure activities. |  |
| Question 3: Would you say you have … | No problems with performing your usual activities? [0] Some problems with performing your usual activities? [1] Are you unable to perform your usual activities? [2] |
| So, would you say you have no problems with performing your usual activities, some problems with performing your usual activities or are you unable to perform your usual activities?  (Note for administrator: tick the appropriate box on the EQ-5D questionnaire) |  |
| PAIN / DISCOMFORT |  |
| Next I'd like to ask you about pain or discomfort. |  |
| Question 4: Would you say you have … | No pain or discomfort? [0] Moderate pain or discomfort? [1] Extreme pain or discomfort? [2] |
| So, would you say you have no pain or discomfort, moderate pain or discomfort or extreme pain or discomfort?  (Note for administrator: tick the appropriate box on the EQ-5D questionnaire) |  |
| ANXIETY / DEPRESSION |  |
| Finally I'd like to ask you about anxiety or depression. |  |
| Question 5: Would you say you are … | Not anxious or depressed? [0] Moderately anxious or depressed? [1] Extremely anxious or depressed? [2] |
| So, would you say you are not anxious or depressed, moderately anxious or depressed or extremely anxious or depressed?  (Note for administrator: tick the appropriate box on the EQ-5D questionnaire) |  |
| # EQ VAS: INTRODUCTION  The best state of health you can imagine is marked 100 (one hundred) at the top of the scale and the worst state you can imagine is marked 0 (zero) at the bottom. |  |
| # EQ VAS: TASK  I would now like you to tell me the point on this scale where you would put your own state of health today. |  |
| Thank you for taking the time to answer these questions. |  |
| HIV Status |  |
| # Instruction to the fieldworker  The following questions should be obtained from the TB treatment records. However, the information might not always be available. We therefore need to confirm the information with our participants.   If the TB records are not available while the participant is with you, please obtain the information from the participant and verify the information with the participant's medical records when you complete the TB TREATMENT RECORDS. |  |
| HIV status | Negative [0] Positive [1] Unknown [2] |
| Date of last HIV test: |  |
| HIV status (Only asked to those who indicated that they are HIV positive) |  |
| CD4 Count: |  |
| HIV THERAPY |  |
| Is the participant on cotrimoxazole?: | No [0] Yes [1] Unknown [2] |
| Is the participant on anti-retroviral therapy (ART)? | No [0] Yes [1] Unknown [2] |
| ART start date: |  |

# MONTH 3 PARTICIPANT FOLLOW-UP QUESTIONS

| SMOKING HISTORY (Only asked to those who smoked at baseline) |  |
| --- | --- |
| Since the start of this study, 3 months ago in (add the month) have you tried quitting smoking tobacco? | No, I continued smoking as before [0] Yes, I reduced my tobacco smoking, but I still smoke regularly [1] Yes, I stopped smoking tobacco completely [2] |
| You indicated that you stopped smoking tobacco altogether. Looking back at the past 3 months, i.e. since the start of the study, how many cigarettes have you smoked in that entire time period? | Not even a puff [0] Between 1 and 5 cigarettes in total [1] More than 5 cigarettes in total [2] |
| Date of breath carbon monoxide reading: (Only asked to those who smoked 5 or less) |  |
| Breath carbon monoxide reading (ppm) (Only asked to those who smoked 5 or less) |  |
| Breath carbon monoxide reading (COHb) (Only asked to those who smoked 5 or less) |  |
| How much have you spent on average *per week* on cigarettes over the past 3 months? R |  |
| INSTRUCTION TO THE FIELDWORKER  This is an unprompted question.  Do not read the list of methods to the participant. Instead, ask the participant to tell you about the methods they used and select the "Yes" answer for the methods that were mentioned.  If the participant did not mention a method on the list of options then you have to select "Yes" to the option "Other" and describe the method provided by the participant.  If the participant indicated that they did use a method in the past to help them stop smoking, then you have to select at least one of the options.  For example, the patient answers that she/he went to the chemist and bought nicotine gum. Then the fieldworker needs to select "Yes" for "Nicotine gum or another nicotine replacement product” from the list of options. |  |
| METHODS TO STOP SMOKING |  |
| Have you ever used any methods in the past to help you stop smoking tobacco in the past 3 months? | No [1] Yes [2] |
| If yes, please describe |  |
| Counselling by a nurse or doctor of counsellor | No [0] Yes [1] |
| Nicotine gum or another nicotine replacement product | No [0] Yes [1] |
| Traditional healer | No [0] Yes [1] |
| Quit helpline | No [0] Yes [1] |
| Other prescribed tobacco cessation treatment (other than Nicotine Replacement therapy) | No [0] Yes [1] |
| Other | No [0] Yes [1] |
| If other, please describe |  |
| How much did you spend (in Rands), out of your pocket, on any of the methods to help you stop smoking in the past 3 months? R |  |
| TRY OR WILL QUIT |  |
| How likely is it that you will TRY TO QUIT smoking completely and permanently in the next three months? | I definitely will not [1] I probably will not [2] I probably will [3] I definitely will [4] |
| How likely is it that you WILL SUCCESSFULLY QUIT smoking completely and permanently in the next three months? | I definitely will not [1] I probably will not [2] I probably will [3] I definitely will [4] |
| Smoking makes me feel bad about myself' | False [0] Mostly false [1] More false than true [2] More True than False [3] Mostly True [4] True [5] |
| In the past month, have you used smokeless tobacco on a daily basis, less than daily basis or not at all | Daily [1] Less than Daily [2] Not at all [0] |
| ALCOHOL QUESTIONS (Only asked to those who drank at baseline) |  |
| I would now like to ask you some questions relating to alcohol use. The questions refer to the PAST 3 MONTHS, i.e. when the study started in (add month). (Record the score for each question in the “Score” column]. |  |
| AUDIT |  |
| Below is a list of questions about your drinking behaviour. Please circle the score (0-4) that best reflects your behaviour. |  |
| How often do you have a drink containing alcohol? | Never [0] Monthly or less [1] 2 to 4 times a month [2] 2 to 3 times a week [3] 4 or more times a week [4] |
| How many drinks containing alcohol do you have on a typical day when you are drinking? (Please note that one drink is equivalent to one can or bottle of beer, cider or cooler, one glass of wine, or one tot of spirits). | 1 or 2 [0] 3 or 4 [1] 5 or 6 [2] 7 to 9 [3] 10 or more [4] |
| How often do you have six or more drinks on one occasion? | Never [0] Less than monthly [1] Monthly [2] Weekly [3] Daily or almost daily [4] |
| How often during the last year have you found that you were not able to stop drinking once you have started? | Never [0] Less than monthly [1] Monthly [2] Weekly [3] Daily or almost daily [4] |
| How often during the last year have you failed to do what was normally expected from you because of drinking? | Never [0] Less than monthly [1] Monthly [2] Weekly [3] Daily or amost daily [4] |
| How often during the last year have you needed a first drink in the morning to get yourself going after a heavy drinking session? | Never [0] Less than monthly [1] Monthly [2] Weekly [3] Daily or almost daily [4] |
| How often during the last year have you had a feeling of guilt or remorse after drinking? | Never [0] Less than monthly [1] Monthly [2] Weekly [3] Daily or almost daily [4] |
| How often during the last year have you been unable to remember what happened the night before because you had been drinking? | Never [0] Less than monthly [1] Monthly [2] Weekly [3] Daily or almost daily [4] |
| Have you or someone else been injured as a result of your drinking? | No [0] Yes, but not in the last year [2] Yes, during the last year [4] |
| Has a relative, friend, or a doctor or another health worker been concerned about your drinking or suggested you cut down? | No [0] Yes, but not in the last year [2] Yes, during the last year [4] |
| The participant's audit score is ____ |  |
| HELP TO STOP DRINKING |  |
| Have you ever used any methods in the past to help you stop drinking alcohol in the past 3 months? | No [0] Yes [1] |
| If yes, please describe |  |
| How much have you spent (in Rands) on average per week on alcohol over the past 3 months? |  |
| TB HISTORY AND MEDICATION ADHERENCE (MODIFIED FOLLOW-UP ACTG) |  |
| TB TREATMENT QUESTIONS |  |
| Are you still taking treatment for TB? | No [0] Yes [1] |
| MISSED DOSES |  |
| ANTI-TB MEDICATION |  |
| Please answer the following questions which asks about TB medications that you might have missed over the last four days: |  |
| # INSTRUCTIONS TO THE FIELDWORKER:   In the following questions regarding the number of doses missed, please note that ONE DOSE is equivalent to the number of pills of a medication taken at a certain time point. For example if taking two pills of medication 'A' in the morning and two in the evening (i.e. two pills twice a day) then, if the two pills in the morning are missed this would be equivalent to ONE DOSE missed. . If the participant missed both the two pills in the morning and the two pills in the evening, then they missed TWO DOSES. |  |
| Do you use the following anti-TB medication? |  |
| RHZE | No [0] Yes [1] |
| RHZE |  |
| If yes, how many doses RHZE did you miss... |  |
| …yesterday? |  |
| …the day before yesterday (2 days ago)? |  |
| …3 days ago? |  |
| …4 days ago? |  |
| RH | No [0] Yes [1] |
| RH |  |
| If yes, how many doses RH did you miss... |  |
| …yesterday? |  |
| …the day before yesterday (2 days ago)? |  |
| …3 days ago? |  |
| …4 days ago? |  |
| During the past 4 days, on how many days have you missed taking all of your doses of TB medications? | None [0] One day [1] Two day [2] Three days [3] Four days [4] |
| Do any of your TB medications have special instructions, such as “take with food” or “on an empty stomach” or “with plenty of fluids?” | No [0] Yes [1] Don't know [2] |
| If “Yes”, how often did you follow those special instructions over the last four days? | Never [0] Some of the time [1] About half of the time [2] Most of the time [3] All of the time [4] |
| Some people find that they forget to take their pills on the weekend days. Did you miss any of your TB medications last weekend— last Saturday or Sunday? | No [0] Yes [1] |
| When was the last time you missed taking any of your TB medications? | Within the past week [1] 1-2 weeks ago [2] 2-4 weeks ago [3] 1-3 Months ago [4] Never skip medication [5] |
| REASONS FOR NOT TAKING MEDICATION |  |
| People may miss taking their medications for various reasons. Here is a list of possible reasons why you may miss taking your medications. How often have you missed taking your TB medications because you: (please tick one box for each option) |  |
| Were away from home? | Never [0] Rarely [1] Sometimes [2] Often [3] |
| Were busy with other things? | Never [0] Rarely [1] Sometimes [2] Often [3] |
| Simply forgot? | Never [0] Rarely [1] Sometimes [2] Often [3] |
| Had too many pills to take? | Never [0] Rarely [1] Sometimes [2] Often [3] |
| Wanted to avoid side effects? | Never [0] Rarely [1] Sometimes [2] Often [3] |
| Did not want others to notice you taking medication? | Never [0] Rarely [1] Sometimes [2] Often [3] |
| Had a change in daily routine? | Never [0] Rarely [1] Sometimes [2] Often [3] |
| Felt like the drug was toxic/harmful? | Never [0] Rarely [1] Sometimes [2] Often [3] |
| Fell asleep/slept through dose time? | Never [0] Rarely [1] Sometimes [2] Often [3] |
| Felt sick or ill? | Never [0] Rarely [1] Sometimes [2] Often [3] |
| Felt depressed/overwhelmed? | Never [0] Rarely [1] Sometimes [2] Often [3] |
| Had problems taking pills at specified times? | Never [0] Rarely [1] Sometimes [2] Often [3] |
| Ran out of pills? | Never [0] Rarely [1] Sometimes [2] Often [3] |
| Felt good? | Never [0] Rarely [1] Sometimes [2] Often [3] |
| Wanted to drink and avoid possible bad reaction with alcohol? | Never [0] Rarely [1] Sometimes [2] Often [3] |
| HIV MEDICATION ADHERENCE (MODIFIED ACTG) |  |
| Are you taking anti-retroviral therapy (ART)? | No [0] Yes [1] |
| ART QUESTIONS (Section only asked to participants who are taking ART ) |  |
| If yes, when did you start taking ART? |  |
| MISSED DOSES |  |
| Please answer the following questions which asks about HIV medications that you might have missed over the last four days: |  |
| INSTRUCTIONS TO THE FIELDWORKER:   In the following questions regarding the number of doses missed, please note that ONE DOSE is equivalent to the number of pills of a medication taken at a certain time point. For example if taking two pills of medication 'A' in the morning and two in the evening (i.e. two pills twice a day) then, if the two pills in the morning are missed this would be equivalent to ONE DOSE missed. . If the participant missed both the two pills in the morning and the two pills in the evening, then they missed TWO DOSES. |  |
| HIV medications |  |
| How many types of medications do you take for HIV? |  |
| INSTRUCTION TO THE FIELDWORKER The following questions should be answered with the assistance of the visual chart which includes the list of HIV medications. |  |
| Do you use the following HIV medications? |  |
| # INSTRUCTION TO THE FIELDWORKER  Show the participant the visual chart and ask them to show you the medications that they are using.  If they show medications on the list then select “Yes” for those medications only. Select “No” for the rest of the medications on the list.  If the participant is using medication that is not listed on the chart then select “Yes” for “My medication is not on this chart/list”.  If they don’t know what they are taking then select “Yes” for “I don't know the name of the medication.” |  |
| ATROIZA (Tenofovir disoproxil fumarate 300 mg, Efavirenz 600 mg, Emtricitabine 200 mg) | No [0] Yes [1] |
| ATROIZA |  |
| How many doses ATROIZA are you supposed to take each day? |  |
| How many doses ATROIZA did you miss... |  |
| …yesterday? |  |
| …the day before yesterday (2 days ago)? |  |
| …3 days ago? |  |
| …4 days ago? |  |
| DUMIVA (Abacavir 600 mg, Lamivudine 300 mg) | No [0] Yes [1] |
| DUMIVA |  |
| How many doses DUMIVA are you supposed to take each day? |  |
| How many doses DUMIVA did you miss... |  |
| …yesterday? |  |
| …the day before yesterday (2 days ago)? |  |
| …3 days ago? |  |
| …4 days ago? |  |
| TENEMINE (Tenofovir disoproxil fumarate 300 mg, Emtricitabine 200 mg) | No [0] Yes [1] |
| TENEMINE |  |
| How many doses TENEMINE are you supposed to take each day? |  |
| How many doses TENEMINE did you miss... |  |
| …yesterday? |  |
| …the day before yesterday (2 days ago)? |  |
| …3 days ago? |  |
| …4 days ago? |  |
| ZOVILAM (Lamivudine 150 mg, Zidovudine 300 mg) | No [0] Yes [1] |
| ZOVILAM |  |
| How many doses ZOVILAM are you supposed to take each day? |  |
| How many doses ZOVILAM did you miss... |  |
| …yesterday? |  |
| …the day before yesterday (2 days ago)? |  |
| …3 days ago? |  |
| …4 days ago? |  |
| KAVIMUN (Abacavir 300 mg) | No [0] Yes [1] |
| KAVIMUN |  |
| How many doses KAVIMUN are you supposed to take each day? |  |
| How many doses KAVIMUN did you miss... |  |
| …yesterday? |  |
| …the day before yesterday (2 days ago)? |  |
| …3 days ago? |  |
| …4 days ago? |  |
| RICOVIR (Tenofovir disoproxil fumarate 300 mg) | No [0] Yes [1] |
| RICOVIR |  |
| How many doses RICOVIR are you supposed to take each day? |  |
| How many doses RICOVIR did you miss… |  |
| …yesterday? |  |
| …the day before yesterday (2 days ago)? |  |
| …3 days ago? |  |
| …4 days ago? |  |
| ZIDOMAT 100mg/300mgZid (Zidovudine 100 mg/300 mg) | No [0] Yes [1] |
| ZIDOMAT |  |
| How many doses ZIDOMAT are you supposed to take each day? |  |
| How many doses ZIDOMAT did you miss... |  |
| …yesterday? |  |
| …the day before yesterday (2 days ago)? |  |
| …3 days ago? |  |
| …4 days ago? |  |
| LAZENA 300 (Lamivudine 300 mg) | No [0] Yes [1] |
| LAZENA |  |
| How many doses LAZENA are you supposed to take each day? |  |
| If yes, how many doses LAZENA 300 did you miss... |  |
| …yesterday? |  |
| …the day before yesterday (2 days ago)? |  |
| …3 days ago? |  |
| …4 days ago? |  |
| EFRIN (Efavirenz 200 mg) | No [0] Yes [1] |
| EFRIN |  |
| How many doses EFRIN are you supposed to take each day? |  |
| How many doses EFRIN did you miss... |  |
| …yesterday? |  |
| …the day before yesterday (2 days ago)? |  |
| …3 days ago? |  |
| …4 days ago? |  |
| EFAMAT 200 mg (Efavirenze 200 mg) | No [0] Yes [1] |
| EFAMAT |  |
| How many doses EFAMAT are you supposed to take each day? |  |
| How many doses EFAMAT did you miss... |  |
| …yesterday? |  |
| …the day before yesterday (2 days ago)? |  |
| …3 days ago? |  |
| …4 days ago? |  |
| ACRIPTAZ 200 mg (Nevirapine 200 mg) | No [0] Yes [1] |
| ACRIPTAZ |  |
| How many doses ACRIPTAZ are you supposed to take each day? |  |
| If yes, how many doses ACRIPTAZ 200 mg did you miss... |  |
| …yesterday? |  |
| …the day before yesterday (2 days ago)? |  |
| …3 days ago? |  |
| …4 days ago? |  |
| My medication is not on this chart/list (Select only if they are not able to identify their medications). [INSERT TRANSLATION] | No [0] Yes [1] |
| NOT ON CHART |  |
| How many doses are you supposed to take each day? |  |
| How many doses did you miss... |  |
| …yesterday? |  |
| …the day before yesterday (2 days ago)? |  |
| …3 days ago? |  |
| …4 days ago? |  |
| I don't know the name of the medication | No [0] Yes [1] |
| DON’T KNOW |  |
| How many doses are you supposed to take each day? |  |
| How many doses did you miss... |  |
| …yesterday? |  |
| …the day before yesterday (2 days ago)? |  |
| …3 days ago? |  |
| …4 days ago? |  |
| During the past 4 days, on how many days have you missed taking all of your doses of HIV medications? | None [0] One day [1] Two day [2] Three days [3] Four days [4] |
| Some HIV medications need to be taken on a schedule, such as “2 times a day” or “3 times a day” or “every 8 hours.” | Never [0] Some of the time [1] About half of the time [2] Most of the time [3] All of the time [4] |
| Do any of your HIV medications have special instructions, such as “take with food” or “on an empty stomach” or “with plenty of fluids?” | No [0] Yes [1] Don't know [2] |
| If “Yes”, how often did you follow those special instructions over the last four days? | Never [0] Some of the time [1] About half of the time [2] Most of the time [3] All of the time [4] |
| Some people find that they forget to take their pills on the weekend days. Did you miss any of your anti-HIV medications last weekend— last Saturday or Sunday? | No [0] Yes [1] |
| When was the last time you missed taking any of your HIV medications? | Within the past week [1] 1-2 weeks ago [2] 2-4 weeks ago [3] 1-3 Months ago [4] Never skip medication [5] |
| REASONS FOR NOT TAKING MEDICATION |  |
| People may miss taking their medications for various reasons. Here is a list of possible reasons why you may miss taking your medications. How often have you missed taking your HIV medications because you: (please tick one box for each option)? |  |
| Were away from home? | Never [0] Rarely [1] Sometimes [2] Often [3] |
| Were busy with other things? | Never [0] Rarely [1] Sometimes [2] Often [3] |
| Simply forgot? | Never [0] Rarely [1] Sometimes [2] Often [3] |
| Had too many pills to take? | Never [0] Rarely [1] Sometimes [2] Often [3] |
| Wanted to avoid side effects? | Never [0] Rarely [1] Sometimes [2] Often [3] |
| Did not want others to notice you taking medication? | Never [0] Rarely [1] Sometimes [2] Often [3] |
| Had a change in daily routine? | Never [0] Rarely [1] Sometimes [2] Often [3] |
| Felt like the drug was toxic/harmful? | Never [0] Rarely [1] Sometimes [2] Often [3] |
| Fell asleep/slept through dose time? | Never [0] Rarely [1] Sometimes [2] Often [3] |
| Felt sick or ill? | Never [0] Rarely [1] Sometimes [2] Often [3] |
| Felt depressed/overwhelmed? | Never [0] Rarely [1] Sometimes [2] Often [3] |
| Had problems taking pills at specified times? | Never [0] Rarely [1] Sometimes [2] Often [3] |
| Ran out of pills? | Never [0] Rarely [1] Sometimes [2] Often [3] |
| Felt good? | Never [0] Rarely [1] Sometimes [2] Often [3] |
| Wanted to drink and avoid possible bad reaction with alcohol? | Never [0] Rarely [1] Sometimes [2] Often [3] |
| CESD 10 |  |
| Now we would like to ask you some questions about how your mood has been lately. For each of the following statements, please check the box that best describes how often you felt or behaved this way during the past week. (CESD 10) |  |
| I was bothered by things that usually don’t bother me. | Rarely or None of the Time (Less than 1 day) [1] Some or a Little of the Time (1-2 days) [2] Occasionally or a Moderate Amount of the Time (3-4 days) [3] Most or All of the Time (5-7 days) [4] |
| I had trouble keeping my mind on what I was doing. | Rarely or None of the Time (Less than 1 day) [1] Some or a Little of the Time (1-2 days) [2] Occasionally or a Moderate Amount of the Time (3-4 days) [3] Most or All of the Time (5-7 days) [4] |
| I felt depressed. | Rarely or None of the Time (Less than 1 day) [1] Some or a Little of the Time (1-2 days) [2] Occasionally or a Moderate Amount of the Time (3-4 days) [3] Most or All of the Time (5-7 days) [4] |
| I felt that everything I did was an effort. | Rarely or None of the Time (Less than 1 day) [1] Some or a Little of the Time (1-2 days) [2] Occasionally or a Moderate Amount of the Time (3-4 days) [3] Most or All of the Time (5-7 days) [4] |
| I felt hopeful about the future. | Rarely or None of the Time (Less than 1 day) [4] Some or a Little of the Time (1-2 days) [3] Occasionally or a Moderate Amount of the Time (3-4 days) [2] Most or All of the Time (5-7 days) [1] |
| I felt fearful. | Rarely or None of the Time (Less than 1 day) [1] Some or a Little of the Time (1-2 days) [2] Occasionally or a Moderate Amount of the Time (3-4 days) [3] Most or All of the Time (5-7 days) [4] |
| My sleep was restless. | Rarely or None of the Time (Less than 1 day) [1] Some or a Little of the Time (1-2 days) [2] Occasionally or a Moderate Amount of the Time (3-4 days) [3] Most or All of the Time (5-7 days) [4] |
| I was happy. | Rarely or None of the Time (Less than 1 day) [4] Some or a Little of the Time (1-2 days) [3] Occasionally or a Moderate Amount of the Time (3-4 days) [2] Most or All of the Time (5-7 days) [1] |
| I felt lonely. | Rarely or None of the Time (Less than 1 day) [1] Some or a Little of the Time (1-2 days) [2] Occasionally or a Moderate Amount of the Time (3-4 days) [3] Most or All of the Time (5-7 days) [4] |
| I could not get “going”. | Rarely or None of the Time (Less than 1 day) [1] Some or a Little of the Time (1-2 days) [2] Occasionally or a Moderate Amount of the Time (3-4 days) [3] Most or All of the Time (5-7 days) [4] |
| HEALTH ECONOMICS |  |
| You should exclude current care that you are receiving in your answers to these questions |  |
| Have you visited any health facility for TB symptoms and/or initial treatment in the past 3 months? | No. [0] Yes [1] |
| FACILITY |  |
| Did you visit a public clinic or health centre? | No [0] Yes [1] |
| Public clinic or facility |  |
| How many times did you visit this facility? |  |
| Tick which health worker you were seen by for the reason of your visit: |  |
| Nurse | No [0] Yes [1] |
| Doctor | No [0] Yes [1] |
| How much did you pay (in Rands) in total including travel per visit? R |  |
| How many hours did it take per visit to travel from home and back? |  |
| Of this time, how many hours did the total consultation for the reason of your visit take? |  |
| Of the total consultation time, how much time (in minutes) did you spend with the doctor? |  |
| Did you visit a public hospital? | No [0] Yes [1] |
| Public hospital |  |
| How many times did you visit this facility? |  |
| Tick which health worker you were seen by for the reason of your visit: |  |
| Nurse | No [0] Yes [1] |
| Doctor | No [0] Yes [1] |
| How much did you pay (in Rands) in total including travel per visit? R |  |
| How many hours did it take per visit to travel from home and back? |  |
| Of this time, how many hours did the total consultation for the reason of your visit take? |  |
| Of the total consultation time, how much time (in minutes) did you spend with the doctor? |  |
| Did you have to stay overnight at any of these visits? | No [0] Yes [1] |
| If Yes, how many nights did you spend per visit? |  |
| EQ-5D-3L |  |
| # INTRODUCTION TO EQ-5D  We are trying to find out what you think about your health. I will first ask you a few brief and simple questions about your own state of health today. I will then ask you to do a rather different task that involves rating your health on a measuring scale. I will explain the tasks fully as I go along but please interrupt me if you do not understand something or if things are not clear to you. Please also remember that there are no right or wrong answers. We are interested here only in your personal view. |  |
| First I am going to read out some questions. Each question has a choice of three answers. Please tell me which answer best describes your own state of health today.  Do not choose more than one answer in each group of questions.  [Note for administrator: it may be necessary to remind the respondent regularly that the timeframe is today] |  |
| MOBILITY |  |
| First I'd like to ask you about mobility. |  |
| Question 1: Would you say you have … | No problems in walking about? [0] Some problems in walking about? [1] Are you confined to bed? [2] |
| So, would you say you have no problems in walking about, some problems in walking about or are you confined to bed?  (Note for administrator: tick the appropriate box on the EQ-5D questionnaire) |  |
| SELF-CARE |  |
| Next I'd like to ask you about self-care. |  |
| Question 2: Would you say you have … | No problems with self-care? [0] Some problems washing or dressing yourself? [1] Are you unable to wash or dress yourself? [2] |
| So, would you say you have no problems with self-care, some problems washing or dressing yourself or are you unable to wash or dress yourself?  (Note for administrator: tick the appropriate box on the EQ-5D questionnaire) |  |
| USUAL ACTIVITIES |  |
| Next I'd like to ask you about usual activities, for example work, study, housework, family or leisure activities. |  |
| Question 3: Would you say you have … | No problems with performing your usual activities? [0] Some problems with performing your usual activities? [1] Are you unable to perform your usual activities? [2] |
| So, would you say you have no problems with performing your usual activities, some problems with performing your usual activities or are you unable to perform your usual activities?  (Note for administrator: tick the appropriate box on the EQ-5D questionnaire) |  |
| PAIN / DISCOMFORT |  |
| Next I'd like to ask you about pain or discomfort. |  |
| Question 4: Would you say you have … | No pain or discomfort? [0] Moderate pain or discomfort? [1] Extreme pain or discomfort? [2] |
| So, would you say you have no pain or discomfort, moderate pain or discomfort or extreme pain or discomfort?  (Note for administrator: tick the appropriate box on the EQ-5D questionnaire) |  |
| ANXIETY / DEPRESSION |  |
| Finally I'd like to ask you about anxiety or depression. |  |
| Question 5: Would you say you are … | Not anxious or depressed? [0] Moderately anxious or depressed? [1] Extremely anxious or depressed? [2] |
| So, would you say you are not anxious or depressed, moderately anxious or depressed or extremely anxious or depressed?  (Note for administrator: tick the appropriate box on the EQ-5D questionnaire) |  |
| # EQ VAS: INTRODUCTION  The best state of health you can imagine is marked 100 (one hundred) at the top of the scale and the worst state you can imagine is marked 0 (zero) at the bottom. |  |
| # EQ VAS: TASK I would now like you to tell me the point on this scale where you would put your own state of health today. |  |
| Thank you for taking the time to answer these questions. |  |

# MONTH 6 PARTICIPANT FOLLOW-UP QUESTIONS

| SMOKING QUESTIONS (Only asked to those who indicated that they smoke at baseline) |  |
| --- | --- |
| QUITTING |  |
| Since the start of this study, 6 months ago in (add the month) have you tried quitting smoking tobacco? | No, I continued smoking as before [0] Yes, I reduced my tobacco smoking, but I still smoke regularly [1] Yes, I stopped smoking tobacco completely [2] |
| You indicated that you stopped smoking tobacco altogether. Looking back at the past 6 months, i.e. since the start of the study, how many cigarettes have you smoked in that entire time period? | Not even a puff [0] Between 1 and 5 cigarettes in total [1] More than 5 cigarettes in total [2] |
| Date of breath carbon monoxide reading:  (Only asked to those who indicated smoking 5 or less cigarettes) |  |
| Breath carbon monoxide reading (ppm)  (Only asked to those who indicated smoking 5 or less cigarettes) |  |
| Breath carbon monoxide reading (COHb)  (Only asked to those who indicated smoking 5 or less cigarettes) |  |
| How much have you spent on average *per week* on cigarettes over the past 3 months? R |  |
| INSTRUCTION TO THE FIELDWORKER  This is an unprompted question.  Do not read the list of methods to the participant. Instead, ask the participant to tell you about the methods they used and select the "Yes" answer for the methods that were mentioned.  If the participant did not mention a method on the list of options then you have to select "Yes" to the option "Other" and describe the method provided by the participant.  If the participant indicated that they did use a method in the past to help them stop smoking, then you have to select at least one of the options.  For example, the patient answers that she/he went to the chemist and bought nicotine gum. Then the fieldworker needs to select "Yes" for "Nicotine gum or another nicotine replacement product” from the list of options. |  |
| HELP TO STOP SMOKING |  |
| Have you ever used any methods in the past to help you stop smoking tobacco in the past 3 months? | No [1] Yes [2] |
| If yes, please describe |  |
| Counselling by a nurse or doctor of counsellor | No [0] Yes [1] |
| Nicotine gum or another nicotine replacement product | No [0] Yes [1] |
| Traditional healer | No [0] Yes [1] |
| Quit helpline | No [0] Yes [1] |
| Other prescribed tobacco cessation treatment (other than Nicotine Replacement therapy) | No [0] Yes [1] |
| Other | No [0] Yes [1] |
| If other, please describe |  |
| How much did you spend (in Rands), out of your pocket, on any of the methods to help you stop smoking in the past 3 months? R |  |
| TRY OR WILL QUIT |  |
| How likely is it that you will TRY TO QUIT smoking completely and permanently in the next three months? | I definitely will not [0] I probably will not [1] I probably will [2] I definitely will [3] |
| How likely is it that you WILL SUCCESSFULLY QUIT smoking completely and permanently in the next three months? | I definitely will not [0] I probably will not [1] I probably will [2] I definitely will [3] |
| Smoking makes me feel bad about myself' | False [0] Mostly false [1] More false than true [2] More True than False [3] Mostly True [4] True [5] |
| In the past month, have you used smokeless tobacco on a daily basis, less than daily basis or not at all | Daily [1] Less than Daily [2] Not at all [0] |
| ALCOHOL QUESTIONS (Only asked to those who indicated at baseline that they drink.) |  |
| I would now like to ask you some questions relating to alcohol use. The questions refer to the PAST 6 MONTHS, i.e. when the study started in (add month). (Record the score for each question in the “Score” column]. |  |
| AUDIT |  |
| Below is a list of questions about your drinking behaviour. Please circle the score (0-4) that best reflects your behaviour.  In the past 12 months: |  |
| How often do you have a drink containing alcohol? | Never [0] Monthly or less [1] 2 to 4 times a month [2] 2 to 3 times a week [3] 4 or more times a week [4] |
| How many drinks containing alcohol do you have on a typical day when you are drinking? (Please note that one drink is equivalent to one can or bottle of beer, cider or cooler, one glass of wine, or one tot of spirits). | 1 or 2 [0] 3 or 4 [1] 5 or 6 [2] 7 to 9 [3] 10 or more [4] |
| How often do you have six or more drinks on one occasion? | Never [0] Less than monthly [1] Monthly [2] Weekly [3] Daily or almost daily [4] |
| How often during the last year have you found that you were not able to stop drinking once you have started? | Never [0] Less than monthly [1] Monthly [2] Weekly [3] Daily or almost daily [4] |
| How often during the last year have you failed to do what was normally expected from you because of drinking? | Never [0] Less than monthly [1] Monthly [2] Weekly [3] Daily or amost daily [4] |
| How often during the last year have you needed a first drink in the morning to get yourself going after a heavy drinking session? | Never [0] Less than monthly [1] Monthly [2] Weekly [3] Daily or almost daily [4] |
| How often during the last year have you had a feeling of guilt or remorse after drinking? | Never [0] Less than monthly [1] Monthly [2] Weekly [3] Daily or almost daily [4] |
| How often during the last year have you been unable to remember what happened the night before because you had been drinking? | Never [0] Less than monthly [1] Monthly [2] Weekly [3] Daily or almost daily [4] |
| Have you or someone else been injured as a result of your drinking? | No [0] Yes, but not in the last year [2] Yes, during the last year [4] |
| Has a relative, friend, or a doctor or another health worker been concerned about your drinking or suggested you cut down? | No [0] Yes, but not in the last year [2] Yes, during the last year [4] |
| The participant's audit score is ____ |  |
| HELP TO STOP DRINKING |  |
| Have you ever used any methods in the past to help you stop drinking alcohol in the past 3 months? | No [0] Yes [1] |
| If yes, please describe |  |
| How much have you spent (in Rands) on average per week on alcohol over the past 3 months? |  |
| TB HISTORY AND MEDICATION ADHERENCE (MODIFIED FOLLOW-UP ACTG) |  |
| TB TREATMENT QUESTIONS |  |
| Are you still taking treatment for TB? | No [0] Yes [1] |
| MISSED DOSES |  |
| ANTI-TB MEDICATION |  |
| Please answer the following questions which asks about TB medications that you might have missed over the last four days: |  |
| INSTRUCTIONS TO THE FIELDWORKER:   In the following questions regarding the number of doses missed, please note that ONE DOSE is equivalent to the number of pills of a medication taken at a certain time point. For example if taking two pills of medication 'A' in the morning and two in the evening (i.e. two pills twice a day) then, if the two pills in the morning are missed this would be equivalent to ONE DOSE missed. . If the participant missed both the two pills in the morning and the two pills in the evening, then they missed TWO DOSES. |  |
| Do you use the following anti-TB medication? |  |
| RHZE | No [0] Yes [1] |
| RHZE |  |
| If yes, how many doses RHZE did you miss... |  |
| …yesterday? |  |
| …the day before yesterday (2 days ago)? |  |
| …3 days ago? |  |
| …4 days ago? |  |
| RH | No [0] Yes [1] |
| RH |  |
| If yes, how many doses RH did you miss... |  |
| …yesterday? |  |
| …the day before yesterday (2 days ago)? |  |
| …3 days ago? |  |
| …4 days ago? |  |
| During the past 4 days, on how many days have you missed taking all of your doses of TB medications? | None [0] One day [1] Two day [2] Three days [3] Four days [4] |
| Do any of your TB medications have special instructions, such as “take with food” or “on an empty stomach” or “with plenty of fluids?” | No [0] Yes [1] Don't know [2] |
| If “Yes”, how often did you follow those special instructions over the last four days? | Never [0] Some of the time [1] About half of the time [2] Most of the time [3] All of the time [4] |
| Some people find that they forget to take their pills on the weekend days. Did you miss any of your TB medications last weekend— last Saturday or Sunday? | No [0] Yes [1] |
| When was the last time you missed taking any of your TB medications? | Within the past week [1] 1-2 weeks ago [2] 2-4 weeks ago [3] 1-3 Months ago [4] Never skip medication [5] |
| REASONS FOR NOT TAKING MEDICATION |  |
| People may miss taking their medications for various reasons. Here is a list of possible reasons why you may miss taking your medications. How often have you missed taking your TB medications because you: (please tick one box for each option) |  |
| Were away from home? | Never [0] Rarely [1] Sometimes [2] Often [3] |
| Were busy with other things? | Never [0] Rarely [1] Sometimes [2] Often [3] |
| Simply forgot? | Never [0] Rarely [1] Sometimes [2] Often [3] |
| Had too many pills to take? | Never [0] Rarely [1] Sometimes [2] Often [3] |
| Wanted to avoid side effects? | Never [0] Rarely [1] Sometimes [2] Often [3] |
| Did not want others to notice you taking medication? | Never [0] Rarely [1] Sometimes [2] Often [3] |
| Had a change in daily routine? | Never [0] Rarely [1] Sometimes [2] Often [3] |
| Felt like the drug was toxic/harmful? | Never [0] Rarely [1] Sometimes [2] Often [3] |
| Fell asleep/slept through dose time? | Never [0] Rarely [1] Sometimes [2] Often [3] |
| Felt sick or ill? | Never [0] Rarely [1] Sometimes [2] Often [3] |
| Felt depressed/overwhelmed? | Never [0] Rarely [1] Sometimes [2] Often [3] |
| Had problems taking pills at specified times? | Never [0] Rarely [1] Sometimes [2] Often [3] |
| Ran out of pills? | Never [0] Rarely [1] Sometimes [2] Often [3] |
| Felt good? | Never [0] Rarely [1] Sometimes [2] Often [3] |
| Wanted to drink and avoid possible bad reaction with alcohol? | Never [0] Rarely [1] Sometimes [2] Often [3] |
| HIV MEDICATION ADHERENCE (MODIFIED ACTG) |  |
| Are you taking anti-retroviral therapy (ART)? | No [0] Yes [1] |
| ART QUESTIONS (Only asked to those who indicated that they are taking ART) |  |
| If yes, when did you start taking ART? |  |
| MISSED DOSES |  |
| Please answer the following questions which asks about HIV medications that you might have missed over the last four days: |  |
| INSTRUCTIONS TO THE FIELDWORKER:   In the following questions regarding the number of doses missed, please note that ONE DOSE is equivalent to the number of pills of a medication taken at a certain time point. For example if taking two pills of medication 'A' in the morning and two in the evening (i.e. two pills twice a day) then, if the two pills in the morning are missed this would be equivalent to ONE DOSE missed. If the participant missed both the two pills in the morning and the two pills in the evening, then they missed TWO DOSES. |  |
| HIV medications |  |
| How many types of medications do you take for HIV? |  |
| INSTRUCTION TO THE FIELDWORKER The following questions should be answered with the assistance of the visual chart which includes the list of HIV medications. |  |
| Do you use the following HIV medications? |  |
| # INSTRUCTION TO THE FIELDWORKER  Show the participant the visual chart and ask them to show you the medications that they are using.  If they show medications on the list then select “Yes” for those medications only. Select “No” for the rest of the medications on the list.  If the participant is using medication that is not listed on the chart then select “Yes” for “My medication is not on this chart/list”.  If they don’t know what they are taking then select “Yes” for “I don't know the name of the medication.” |  |
| ATROIZA (Tenofovir disoproxil fumarate 300 mg, Efavirenz 600 mg, Emtricitabine 200 mg) | No [0] Yes [1] |
| ATROIZA |  |
| How many doses ATROIZA are you supposed to take each day? |  |
| How many doses ATROIZA did you miss... |  |
| …yesterday? |  |
| …the day before yesterday (2 days ago)? |  |
| …3 days ago? |  |
| …4 days ago? |  |
| DUMIVA (Abacavir 600 mg, Lamivudine 300 mg) | No [0] Yes [1] |
| DUMIVA |  |
| How many doses DUMIVA are you supposed to take each day? |  |
| How many doses DUMIVA did you miss... |  |
| …yesterday? |  |
| …the day before yesterday (2 days ago)? |  |
| …3 days ago? |  |
| …4 days ago? |  |
| TENEMINE (Tenofovir disoproxil fumarate 300 mg, Emtricitabine 200 mg) | No [0] Yes [1] |
| TENEMINE |  |
| How many doses TENEMINE are you supposed to take each day? |  |
| How many doses TENEMINE did you miss... |  |
| …yesterday? |  |
| …the day before yesterday (2 days ago)? |  |
| …3 days ago? |  |
| …4 days ago? |  |
| ZOVILAM (Lamivudine 150 mg, Zidovudine 300 mg) | No [0] Yes [1] |
| ZOVILAM |  |
| How many doses ZOVILAM are you supposed to take each day? |  |
| If yes, how many doses ZOVILAM did you miss... |  |
| …yesterday? |  |
| …the day before yesterday (2 days ago)? |  |
| …3 days ago? |  |
| …4 days ago? |  |
| KAVIMUN (Abacavir 300 mg) | No [0] Yes [1] |
| KAVIMUN |  |
| How many doses KAVIMUN are you supposed to take each day? |  |
| How many doses KAVIMUN did you miss... |  |
| …yesterday? |  |
| …the day before yesterday (2 days ago)? |  |
| …3 days ago? |  |
| …4 days ago? |  |
| RICOVIR (Tenofovir disoproxil fumarate 300 mg) | No [0] Yes [1] |
| RICOVIR |  |
| How many doses RICOVIR are you supposed to take each day? |  |
| How many doses RICOVIR did you miss... |  |
| …yesterday? |  |
| …the day before yesterday (2 days ago)? |  |
| …3 days ago? |  |
| …4 days ago? |  |
| ZIDOMAT 100mg/300mg (Zidovudine 100 mg/300 mg) | No [0] Yes [1] |
| ZIDOMAT |  |
| How many doses ZIDOMAT are you supposed to take each day? |  |
| How many doses ZIDOMAT did you miss... |  |
| …yesterday? |  |
| …the day before yesterday (2 days ago)? |  |
| …3 days ago? |  |
| …4 days ago? |  |
| LAZENA 300 (Lamivudine 300 mg) | No [0] Yes [1] |
| LAZENA |  |
| How many doses LAZENA are you supposed to take each day? |  |
| How many doses LAZENA did you miss... |  |
| …yesterday? |  |
| …the day before yesterday (2 days ago)? |  |
| …3 days ago? |  |
| …4 days ago? |  |
| EFRIN (Efavirenz 200 mg) | No [0] Yes [1] |
| EFRIN |  |
| How many doses EFRIN are you supposed to take each day? |  |
| How many doses EFRIN did you miss... |  |
| …yesterday? |  |
| …the day before yesterday (2 days ago)? |  |
| …3 days ago? |  |
| …4 days ago? |  |
| EFAMAT 200 mg (Efavirenze 200 mg) | No [0] Yes [1] |
| EFAMAT |  |
| How many doses EFAMAT are you supposed to take each day? |  |
| How many doses EFAMAT did you miss... |  |
| …yesterday? |  |
| …the day before yesterday (2 days ago)? |  |
| …3 days ago? |  |
| …4 days ago? |  |
| ACRIPTAZ 200 mg (Nevirapine 200 mg) | No [0] Yes [1] |
| ACRIPTAZ |  |
| How many doses ACRIPTAZ are you supposed to take each day? |  |
| How many doses ACRIPTAZ did you miss... |  |
| …yesterday? |  |
| …the day before yesterday (2 days ago)? |  |
| …3 days ago? |  |
| …4 days ago? |  |
| My medication is not on this chart/list (Select only if they are not able to identify their medications). | No [0] Yes [1] |
| NOT ON CHART |  |
| How many doses are you supposed to take each day? |  |
| How many doses did you miss... |  |
| …yesterday? |  |
| …the day before yesterday (2 days ago)? |  |
| …3 days ago? |  |
| …4 days ago? |  |
| I don't know the name of the medication | No [0] Yes [1] |
| DON’T KNOW |  |
| How many doses are you supposed to take each day? |  |
| How many doses did you miss... |  |
| …yesterday? |  |
| …the day before yesterday (2 days ago)? |  |
| …3 days ago? |  |
| …4 days ago? |  |
| During the past 4 days, on how many days have you missed taking all of your doses of HIV medications? | None [0] One day [1] Two day [2] Three days [3] Four days [4] |
| Some HIV medications need to be taken on a schedule, such as “2 times a day” or “3 times a day” or “every 8 hours.” How closely did you follow your specific schedule over the last four days? | Never [0] Some of the time [1] About half of the time [2] Most of the time [3] All of the time [4] |
| Do any of your HIV medications have special instructions, such as “take with food” or “on an empty stomach” or “with plenty of fluids?” | No [0] Yes [1] Don't know [2] |
| If “Yes”, how often did you follow those special instructions over the last four days? | Never [0] Some of the time [1] About half of the time [2] Most of the time [3] All of the time [4] |
| Some people find that they forget to take their pills on the weekend days. Did you miss any of your anti-HIV medications last weekend— last Saturday or Sunday? | No [0] Yes [1] |
| When was the last time you missed taking any of your HIV medications? | Within the past week [1] 1-2 weeks ago [2] 2-4 weeks ago [3] 1-3 Months ago [4] Never skip medication [5] |
| REASONS FOR NOT TAKING MEDICATION |  |
| People may miss taking their medications for various reasons. Here is a list of possible reasons why you may miss taking your medications. How often have you missed taking your HIV medications because you: (please tick one box for each option)? |  |
| Were away from home? | Never [0] Rarely [1] Sometimes [2] Often [3] |
| Were busy with other things? | Never [0] Rarely [1] Sometimes [2] Often [3] |
| Simply forgot? | Never [0] Rarely [1] Sometimes [2] Often [3] |
| Had too many pills to take? | Never [0] Rarely [1] Sometimes [2] Often [3] |
| Wanted to avoid side effects? | Never [0] Rarely [1] Sometimes [2] Often [3] |
| Did not want others to notice you taking medication? | Never [0] Rarely [1] Sometimes [2] Often [3] |
| Had a change in daily routine? | Never [0] Rarely [1] Sometimes [2] Often [3] |
| Felt like the drug was toxic/harmful? | Never [0] Rarely [1] Sometimes [2] Often [3] |
| Fell asleep/slept through dose time? | Never [0] Rarely [1] Sometimes [2] Often [3] |
| Felt sick or ill? | Never [0] Rarely [1] Sometimes [2] Often [3] |
| Felt depressed/overwhelmed? | Never [0] Rarely [1] Sometimes [2] Often [3] |
| Had problems taking pills at specified times? | Never [0] Rarely [1] Sometimes [2] Often [3] |
| Ran out of pills? | Never [0] Rarely [1] Sometimes [2] Often [3] |
| Felt good? | Never [0] Rarely [1] Sometimes [2] Often [3] |
| Wanted to drink and avoid possible bad reaction with alcohol? | Never [0] Rarely [1] Sometimes [2] Often [3] |
| CESD 10 |  |
| Now we would like to ask you some questions about how your mood has been lately. For each of the following statements, please check the box that best describes how often you felt or behaved this way during the past week. (CESD 10) |  |
| I was bothered by things that usually don’t bother me. | Rarely or None of the Time (Less than 1 day) [1] Some or a Little of the Time (1-2 days) [2] Occasionally or a Moderate Amount of the Time (3-4 days) [3] Most or All of the Time (5-7 days) [4] |
| I had trouble keeping my mind on what I was doing. | Rarely or None of the Time (Less than 1 day) [1] Some or a Little of the Time (1-2 days) [2] Occasionally or a Moderate Amount of the Time (3-4 days) [3] Most or All of the Time (5-7 days) [4] |
| I felt depressed. | Rarely or None of the Time (Less than 1 day) [1] Some or a Little of the Time (1-2 days) [2] Occasionally or a Moderate Amount of the Time (3-4 days) [3] Most or All of the Time (5-7 days) [4] |
| I felt that everything I did was an effort. | Rarely or None of the Time (Less than 1 day) [1] Some or a Little of the Time (1-2 days) [2] Occasionally or a Moderate Amount of the Time (3-4 days) [3] Most or All of the Time (5-7 days) [4] |
| I felt hopeful about the future. | Rarely or None of the Time (Less than 1 day) [4] Some or a Little of the Time (1-2 days) [3] Occasionally or a Moderate Amount of the Time (3-4 days) [2] Most or All of the Time (5-7 days) [1] |
| I felt fearful. | Rarely or None of the Time (Less than 1 day) [1] Some or a Little of the Time (1-2 days) [2] Occasionally or a Moderate Amount of the Time (3-4 days) [3] Most or All of the Time (5-7 days) [4] |
| My sleep was restless. | Rarely or None of the Time (Less than 1 day) [1] Some or a Little of the Time (1-2 days) [2] Occasionally or a Moderate Amount of the Time (3-4 days) [3] Most or All of the Time (5-7 days) [4] |
| I was happy. | Rarely or None of the Time (Less than 1 day) [4] Some or a Little of the Time (1-2 days) [3] Occasionally or a Moderate Amount of the Time (3-4 days) [2] Most or All of the Time (5-7 days) [1] |
| I felt lonely. | Rarely or None of the Time (Less than 1 day) [1] Some or a Little of the Time (1-2 days) [2] Occasionally or a Moderate Amount of the Time (3-4 days) [3] Most or All of the Time (5-7 days) [4] |
| I could not get “going”. | Rarely or None of the Time (Less than 1 day) [1] Some or a Little of the Time (1-2 days) [2] Occasionally or a Moderate Amount of the Time (3-4 days) [3] Most or All of the Time (5-7 days) [4] |
| HEALTH ECONOMICS |  |
| You should exclude current care that you are receiving in your answers to these questions |  |
| Have you visited any health facility for TB symptoms and/or initial treatment in the past 3 months (excluding current care)? | No [0] Yes [1] |
| FACILITY |  |
| Did you visit a public clinic or health centre? | No [0] Yes [1] |
| Public clinic or facility (ambulant) |  |
| How many times did you visit this facility? |  |
| Tick which health worker you were seen by for the reason of your visit: |  |
| Nurse | No [0] Yes [1] |
| Doctor | No [0] Yes [1] |
| How much did you pay (in Rands) in total including travel per visit? R |  |
| How many hours did it take per visit to travel from home and back? |  |
| Of this time, how many hours did the total consultation for the reason of your visit take? |  |
| Of the total consultation time, how much time (in minutes) did you spend with the doctor? |  |
| Did you visit a public hospital? | No [0] Yes [1] |
| Public hospital |  |
| How many times did you visit this facility? |  |
| Tick which health worker you were seen by for the reason of your visit: |  |
| Nurse | No [0] Yes [1] |
| Doctor | No [0] Yes [1] |
| How much did you pay (in Rands) in total including travel per visit? R |  |
| How many hours did it take per visit to travel from home and back? |  |
| Of this time, how many hours did the total consultation for the reason of your visit take (i.e. from the time a nurse or doctor saw you for the reason of your visit to the time that you no longer had to see them again)? |  |
| Of the total consultation time, how much time (in minutes) did you spend with the doctor (if you answered “Yes” in 1.2.2.2) |  |
| Did you have to stay overnight at any of these visits? | No [0] Yes [1] |
| If Yes, how many nights did you spend per visit? |  |
| EQ-5D-3L |  |
| # INTRODUCTION TO EQ-5D  We are trying to find out what you think about your health. I will first ask you a few brief and simple questions about your own state of health today. I will then ask you to do a rather different task that involves rating your health on a measuring scale. I will explain the tasks fully as I go along but please interrupt me if you do not understand something or if things are not clear to you. Please also remember that there are no right or wrong answers. We are interested here only in your personal view. |  |
| First I am going to read out some questions. Each question has a choice of three answers. Please tell me which answer best describes your own state of health today.  Do not choose more than one answer in each group of questions.  [Note for administrator: it may be necessary to remind the respondent regularly that the timeframe is today] |  |
| MOBILITY |  |
| First I'd like to ask you about mobility. |  |
| Question 1: Would you say you have … | No problems in walking about? [0] Some problems in walking about? [1] Are you confined to bed? [2] |
| So, would you say you have no problems in walking about, some problems in walking about or are you confined to bed?  (Note for administrator: tick the appropriate box on the EQ-5D questionnaire) |  |
| SELF-CARE |  |
| Next I'd like to ask you about self-care. |  |
| Question 2: Would you say you have … | No problems with self-care? [0] Some problems washing or dressing yourself? [1] Are you unable to wash or dress yourself? [2] |
| So, would you say you have no problems with self-care, some problems washing or dressing yourself or are you unable to wash or dress yourself?  (Note for administrator: tick the appropriate box on the EQ-5D questionnaire) |  |
| USUAL ACTIVITIES |  |
| Next I'd like to ask you about usual activities, for example work, study, housework, family or leisure activities. |  |
| Question 3: Would you say you have … | No problems with performing your usual activities? [0] Some problems with performing your usual activities? [1] Are you unable to perform your usual activities? [2] |
| So, would you say you have no problems with performing your usual activities, some problems with performing your usual activities or are you unable to perform your usual activities?  (Note for administrator: tick the appropriate box on the EQ-5D questionnaire) |  |
| PAIN / DISCOMFORT |  |
| Next I'd like to ask you about pain or discomfort. |  |
| Question 4: Would you say you have … | No pain or discomfort? [0] Moderate pain or discomfort? [1] Extreme pain or discomfort? [2] |
| So, would you say you have no pain or discomfort, moderate pain or discomfort or extreme pain or discomfort?  (Note for administrator: tick the appropriate box on the EQ-5D questionnaire) |  |
| ANXIETY / DEPRESSION |  |
| Finally I'd like to ask you about anxiety or depression. |  |
| Question 5: Would you say you are … | Not anxious or depressed? [0] Moderately anxious or depressed? [1] Extremely anxious or depressed? [2] |
| So, would you say you are not anxious or depressed, moderately anxious or depressed or extremely anxious or depressed?  (Note for administrator: tick the appropriate box on the EQ-5D questionnaire) |  |
| # EQ VAS: INTRODUCTION  The best state of health you can imagine is marked 100 (one hundred) at the top of the scale and the worst state you can imagine is marked 0 (zero) at the bottom. |  |
| # EQ VAS: TASK I would now like you to tell me the point on this scale where you would put your own state of health today. |  |
| Thank you for taking the time to answer these questions. |  |

# TB TREATMENT RECORDS

| HIV Status |  |
| --- | --- |
| HIV status | Negative [0] Positive [1] Unknown [2] |
| Date of last HIV test: |  |
| HIV status (Only asked to those who indicated that they are HIV positive) |  |
| CD4 Count: |  |
| HIV THERAPY |  |
| Is the participant on cotrimoxazole?: | No [0] Yes [1] Unknown [2] |
| Is the participant on anti-retroviral therapy (ART)? | No [0] Yes [1] Unknown [2] |
| ART start date: |  |
| TB HISTORY |  |
| Patient category: | New patient [1] Relapse [2] Re-treatment after default [3] Re-treatment after failure [4] Other (please give details) [5] Missing/Not recorded [0] |
| If other, please specify. |  |
| Site of disease: | Pulmonary only (ICD-10 A15) [1] Pulmonary and Extra Pulmonary (ICD-10 A17-A19) [0] Missing/Not recorded [2] |
| If Extra Pulmonary TB, please specify site. |  |
| Pre-treatment smear results |  |
| How many pre-treatment smear results are recorded? | One [1] Two [2] Missing/not recorded [0] |
| Smear test 1 |  |
| Pre-treatment smear test date (Smear test 1): |  |
| Pre-treatment smear microscopy result (Smear test 1): | Positive [1] Negative [0] Missing/Not recorded [3] |
| Smear test 2 |  |
| Pre-treatment smear test date (Smear test 1): |  |
| Pre-treatment smear microscopy result (Smear test 1): | Positive [1] Negative [0] Missing/Not recorded [3] |
| Gene XPert results |  |
| How many Gene XPert results have been recorded? | One [1] Two [2] Missing/not recorded [0] |
| Gene Xpert (Result 1) |  |
| Date of Gene Xpert (Result 1) (dd/mm/yyyy): |  |
| Gene Xpert result (Result 1): |  |
| Result: | Positive [1] Negative [2] Unsuccessful [0] Missing/Not recorded [3] |
| Drug susceptibility result (rifampicin): | Resistant [1] Sensitive [2] Unsuccessful [0] Missing/Not recorded [3] |
| Gene Xpert (Result 2) |  |
| Date of Gene Xpert (Result 2) (dd/mm/yyyy): |  |
| Gene Xpert result (Result 2): |  |
| Result: | Positive [1] Negative [2] Unsuccessful [0] Missing/Not recorded [3] |
| Drug susceptibility result (rifampicin): | Resistant [1] Sensitive [2] Unsuccessful [0] Missing/Not recorded [3] |
| Culture results |  |
| How many culture results have been recorded on the TB Treatment record? | One [1] Two [2] Missing/not recorded [0] |
| Culture Result 1 |  |
| Date of culture (Result 1): |  |
| Culture result (Result 1): |  |
| Result: | Positive [1] Negative [2] Contaminated [0] Missing/Not recorded [3] |
| Drug susceptibility result (rifampicin): | Resistant [1] Sensitive [2] Missing/Not recorded [3] |
| Drug susceptibility result (isoniazid): | Resistant [1] Sensitive [2] Missing/Not recorded [3] |
| Culture Result 2 |  |
| Date of culture (Result 2): |  |
| Culture result (Result 2): |  |
| Result: | Positive [1] Negative [2] Contaminated [0] Missing/Not recorded [3] |
| Drug susceptibility result (rifampicin): | Resistant [1] Sensitive [2] Missing/Not recorded [0] |
| Drug susceptibility result (isoniazid): | Resistant [1] Sensitive [2] Missing/Not recorded [0] |
| Chest radiograph result: |  |
| TB TREATMENT |  |
| TREATMENT REGIMEN | Regimen 1 [1] Regimen 3A [2] Regimen 3B [3] Other [4] |
| If other, specify. |  |
| TB treatment start date: |  |
| INTENSIVE PHASE (IP) |  |
| Please add the number of tablets prescribed for the INTENSIVE PHASE (IP).  Write 0 if nothing is prescribed. |  |
| RHZE (150/75/400/275) |  |
| RH (60/60) |  |
| H |  |
| Z |  |
| E |  |
| S |  |
| CO-MORBIDITIES |  |
| Please copy from “Medical History” section of TB Treatment Record |  |
| Does the patient have any of the following conditions? |  |
| Hypertension | No [0] Yes [1] Not available [3] |
| If yes, please specify current medications.  Write 0 if nothing is prescribed. |  |
| If yes, please specify current medications.  Write "Nothing" if no information is provided. |  |
| 1 |  |
| 2 |  |
| 3 |  |
| 4 |  |
| Diabetes | No [0] Yes [1] Not available [3] |
| If yes, please specify current medications. |  |
| If yes, please specify current medications.  Write "Nothing" if no information is provided. |  |
| 1 |  |
| 2 |  |
| 3 |  |
| 4 |  |
| Epilepsy | No [0] Yes [1] Not available [3] |
| If yes, please specify current medications. |  |
| If yes, please specify current medications.  Write "Nothing" if no information is provided. |  |
| 1 |  |
| 2 |  |
| 3 |  |
| 4 |  |
| Mental illness | No [0] Yes [1] Not available [3] |
| If yes, please specify current medications. |  |
| If yes, please specify current medications.  Write "Nothing" if no information is provided. |  |
| 1 |  |
| 2 |  |
| 3 |  |
| 4 |  |
| Liver disease | No [0] Yes [1] Not available [3] |
| If yes, please specify current medications |  |
| If yes, please specify current medications.  Write "Nothing" if no information is provided. |  |
| 1 |  |
| 2 |  |
| 3 |  |
| 4 |  |
| Renal insufficiency | No [0] Yes [1] Not available [3] |
| If yes, please specify current medications. |  |
| If yes, please specify current medications.  Write "Nothing" if no information is provided. |  |
| 1 |  |
| 2 |  |
| 3 |  |
| 4 |  |
| Allergies | No [0] Yes [1] Not available [3] |
| Please specify |  |
| Please specify |  |
| 1 |  |
| 2 |  |
| 3 |  |
| 4 |  |
| If yes, please specify current medications. |  |
| If yes, please specify current medications.  Write "Nothing" if no information is provided. |  |
| 1 |  |
| 2 |  |
| 3 |  |
| 4 |  |
| Other | No [0] Yes [1] Not available [3] |
| Please specify |  |
| Please specify |  |
| 1 |  |
| 2 |  |
| 3 |  |
| 4 |  |
| If yes, please specify current medications. |  |
| If yes, please specify current medications.  Write "Nothing" if no information is provided. |  |
| 1 |  |
| 2 |  |
| 3 |  |
| 4 |  |
| Medications Missed |  |
| With reference to the TB Treatment record, please record any medications missed by participants:  Write 999999 if this value wasn't recorded. |  |
| Month 1: Number of doses missed |  |
| Month 2: Number of doses missed |  |
| Month 3: Number of doses missed |  |
| Month 4: Number of doses missed |  |
| Month 5: Number of doses missed |  |
| Month 6: Number of doses missed |  |
| 2-Month Smear microscopy |  |
| How many results are recorded on the TB Treatment Record for smear at 2 months? | One [1] Two [2] Missing/not recorded [0] |
| Smear test 1 |  |
| 2 Month Smear microscopy test date (Result 1): |  |
| 2 Month smear microscopy result (Result 1): | Negative [0] Positive [1] Missing/not recorded [2] |
| Smear test 2 |  |
| 2 Month Smear microscopy test date (Result 2) |  |
| 2 Month smear microscopy result (Result 2): | Negative [0] Positive [1] Missing/not recorded [2] |
| 3 Month Smear microscopy |  |
| How many results are recorded on the TB Treatment Record for smear at 3 months? | One [1] Two [2] Missing/not recorded [0] |
| Smear test 1 |  |
| 3 Month Smear microscopy test date (Result 1): |  |
| 3 Month smear microscopy result (Result 1): | Negative [0] Positive [1] Missing/not recorded [2] |
| Smear test 2 |  |
| 3 Month Smear microscopy test date (Result 2) : |  |
| 3 Month smear microscopy result (Result 2): | Negative [0] Positive [1] Missing/not recorded [2] |
| 6-Month Smear microscopy |  |
| How many results are recorded on the TB Treatment Record for smear at 6 | One [1] Two [2] Missing/not recorded [0] |
| Smear test 1 |  |
| 6 Month Smear microscopy test date (Result 1): |  |
| 6 Month smear microscopy result (Result 1): | Negative [0] Positive [1] Missing/not recorded [2] |
| Smear test 2 |  |
| 6 Month Smear microscopy test date (Result 2) |  |
| 6 Month smear microscopy result (Result 2): | Negative [0] Positive [1] Missing/not recorded [2] |
| Repeat culture |  |
| Date of repeat culture: |  |
| Culture result: |  |
| Result: | Contaminated [0] Positive [1] Negative [2] Missing/not recorded [3] |
| Drug susceptibility result (rifampicin): | Resistant [1] Sensitive [2] Missing/not recorded [0] |
| Drug susceptibility result (isoniazid): | Resistant [1] Sensitive [2] Missing/not recorded [0] |
| Treatment outcome (This information should be gained from the TB Treatment Record. The TB register should be consulted where information is missing. Attempts should be made to contact any patients lost to follow up): | Cured [0] Treatment Completed [1] ‘Defaulted’ Treatment [2] Failed Treatment [3] Drug resistance developed in the course of treatment [4] Died [5] Transfer out [6] Unkown [7] |
| Treatment outcome date: |  |
| Date of Completion: |  |
