# Supplementary material for: ImPROving TB outcomes by modifying LIFE-style behaviours through a brief motivational intervention followed by short text messages (ProLife): study protocol for a randomised controlled trial
Source: Trials. 2019 Jul 26;20:457. doi: 10.1186/s13063-019-3551-9 (PMC6660690; doi:10.1186/s13063-019-3551-9)
Supplement: Supplementary file 3 — Counselling Activities Reporting Form. (DOC 103 kb) [file 13063_2019_3551_MOESM3_ESM.doc]

**Im*pro*ving TB outcomes by modifying *life*-style behaviours**

**through a brief motivational intervention (PROLIFE)**

- **COUNSELLING ACTIVITIES REPORTING FORM –**

***THIS FORM MUST BE COMPLETED AFTER EACH COUNSELLING SESSION***

| **Name of Counsellor:** |  | **Clinic:** |  |
| --- | --- | --- | --- |
| **Participant Study Number:** |  | **Date:** |  |
| **Gender of Client:** |  | **Age of Participant:** |  |

**Session (circle, as applicable): 1 2 3**

**SECTION 1:**

1. Did you complete a full MI session?

| No, not at all | 1 |
| --- | --- |
| Yes, partially | 2 |
| Yes, completely | 3 |

1. Which behaviour did you focus on today?

| Treatment adherence | 1 |
| --- | --- |
| Tobacco use | 2 |
| Alcohol consumption | 3 |
| Other | 4 |

1. Behaviour for which Importance and Confidence were rated:

| Treatment adherence | 1 |
| --- | --- |
| Tobacco use | 2 |
| Alcohol consumption | 3 |

1. Importance score (circle one):

1 2 3 4 5 6 7 8 9 10

Very low Very high

1. Confidence score (circle one):

1 2 3 4 5 6 7 8 9 10

Very low Very high

1. Did you discuss what it would take for the participant to move up/increase their Importance/Confidence score?

| Yes | 1 |
| --- | --- |
| No | 0 |

1. Did you discuss the reasons behind their Importance/Confidence score?

| Yes | 1 |
| --- | --- |
| No | 0 |

1. Goal that patient plans to achieve for the next visit:

|  |
| --- |

1. Did you refer this participant?

| Yes | 1 |
| --- | --- |
| No | 0 |

1. What issue did you refer the participant for?

|  |
| --- |

1. Participant’s progress on previous goal:

| N/A: Today is the first session | 0 |
| --- | --- |
| No goal set at last visit | 1 |
| No progress on goal | 2 |
| Goal partially achieved | 3 |
| Goal fully achieved | 4 |

1. Barriers to achieving the goal:

|  |
| --- |
|  |
|  |
|  |
|  |

**SECTION 2**

*YOUR EXPERIENCE OF CONDUCTING MI WITH THE PARTICIPANT*

**How difficult or easy did you find it to use each of the following aspects of MI with the client during this session?**

1. Agenda mapping

| Easy | 0 |
| --- | --- |
| Neither easy/nor difficult | 1 |
| Difficult | 2 |

1. Asking open-ended questions

| Easy | 0 |
| --- | --- |
| Neither easy/nor difficult | 1 |
| Difficult | 2 |

1. Affirming the participant (saying something positive/complementary)

| Easy | 0 |
| --- | --- |
| Neither easy/nor difficult | 1 |
| Difficult | 2 |

1. Reflective listening

| Easy | 0 |
| --- | --- |
| Neither easy/nor difficult | 1 |
| Difficult | 2 |

1. Summarizing

| Easy | 0 |
| --- | --- |
| Neither easy/nor difficult | 1 |
| Difficult | 2 |

1. Keeping direction

| Easy | 0 |
| --- | --- |
| Neither easy/nor difficult | 1 |
| Difficult | 2 |

1. Asking for permission before giving advice/information

| Easy | 0 |
| --- | --- |
| Neither easy/nor difficult | 1 |
| Difficult | 2 |

1. Emphasizing the client’s control (autonomy, freedom of choice, ability to decide).

| Easy | 0 |
| --- | --- |
| Neither easy/nor difficult | 1 |
| Difficult | 2 |

1. Supporting the client’s self-efficacy.

| Easy | 0 |
| --- | --- |
| Neither easy/nor difficult | 1 |
| Difficult | 2 |

1. Evoking change talk

| Easy | 0 |
| --- | --- |
| Neither easy/nor difficult | 1 |
| Difficult | 2 |

1. Responding to sustain talk

| Easy | 0 |
| --- | --- |
| Neither easy/nor difficult | 1 |
| Difficult | 2 |

1. Using the decisional balance

| Easy | 0 |
| --- | --- |
| Neither easy/nor difficult | 1 |
| Difficult | 2 |

1. Using the readiness ruler

| Easy | 0 |
| --- | --- |
| Neither easy/nor difficult | 1 |
| Difficult | 2 |

1. Assessing importance

| Easy | 0 |
| --- | --- |
| Neither easy/nor difficult | 1 |
| Difficult | 2 |

1. Assessing confidence

| Easy | 0 |
| --- | --- |
| Neither easy/nor difficult | 1 |
| Difficult | 2 |

**SECTION 3**

Please use this space to provide any additional information you would like to share regarding any aspects of this counselling experience (e.g. logistical or personal issues etc.)

Challenges

|  |
| --- |
|  |
|  |
|  |
|  |

Successes

|  |
| --- |
|  |
|  |
|  |
|  |

**General Comments**

|  |
| --- |
|  |
|  |
|  |
|  |
